# Active nuclear import and passive nuclear export are the primary determinants of TDP-43 localization

**DOI:** 10.1038/s41598-018-25008-4

**Published:** 2018-05-04

**Authors:** Emile S. Pinarbasi, Tolga Cağatay, Ho Yee Joyce Fung, Ying C. Li, Yuh Min Chook, Philip J. Thomas

**Affiliations:** 1 Department of Physiology, UT Southwestern Medical Center at Dallas, Dallas, TX 75390 USA; 2 Medical Scientist Training Program, UT Southwestern Medical Center at Dallas, Dallas, TX 75390 USA; 3 Department of Pharmacology, UT Southwestern Medical Center at Dallas, Dallas, TX 75390 USA; 4 Department of Neuroscience, UT Southwestern Medical Center at Dallas, Dallas, TX 75390 USA

## Abstract

ALS (Amyotrophic Lateral Sclerosis) is a neurodegenerative disease characterized by the redistribution of the RNA binding protein TDP-43 in affected neurons: from predominantly nuclear to aggregated in the cytosol. However, the determinants of TDP-43 localization and the cellular insults that promote redistribution are incompletely understood. Here, we show that the putative Nuclear Export Signal (NES) is not required for nuclear egress of TDP-43. Moreover, when the TDP-43 domain which contains the putative NES is fused to a reporter protein, YFP, the presence of the NES is not sufficient to mediate nuclear exclusion of the fusion protein. We find that the previously studied “∆NES” mutant, in which conserved hydrophobic residues are mutated to alanines, disrupts both solubility and splicing function. We further show that nuclear export of TDP-43 is independent of the exportin XPO1. Finally, we provide evidence that nuclear egress of TDP-43 is size dependent; nuclear export of dTomato TDP-43 is significantly impaired compared to Flag TDP-43. Together, these results suggest nuclear export of TDP-43 is predominantly driven by passive diffusion.

## Introduction

Amyotrophic Lateral Sclerosis (ALS) is an adult-onset neurodegenerative disease which preferentially targets motor neurons, causing muscle weakness and eventually paralysis^1^. ALS is rapidly progressive and ultimately fatal^1^. While the mechanisms underlying the degeneration of motor neurons remain unclear, the RNA-binding protein TDP-43 has emerged as a key player in ALS pathogenesis.

TDP-43 is ubiquitously expressed and highly conserved, with homologs in *C elegans*, *D melanogaster*, mouse, and rat^2^. Underscoring its importance, germline loss of TDP-43 is embryonic lethal for mice and causes dramatic locomotive defects in *D melanogaster*^3–5^. Many functions have been ascribed to TDP-43, but the best described is its role in splicing, especially in repressing inclusion of cryptic exons^6–10^.

Multiple lines of evidence implicate TDP-43 aggregation in the pathogenesis of ALS. First, the characteristic histopathology of ALS: affected neurons contain cytosolic protein aggregates which are composed of ubiquitinated TDP-43, with only rare exceptions^11,12^. Notably, TDP-43 aggregation is accompanied by loss of soluble TDP-43 from the nucleus^11^. Second, point mutations in TDP-43 are a rare cause of familial ALS; many of these mutations have been demonstrated to increase the propensity of TDP-43 to aggregate^13,14^. Finally, several animal models which replicate the ALS-linked aggregation and redistribution of TDP-43 in motor neurons demonstrate the progressive muscle weakness and loss of spinal cord mass seen in patients^15,16^.

**Figure 1:**
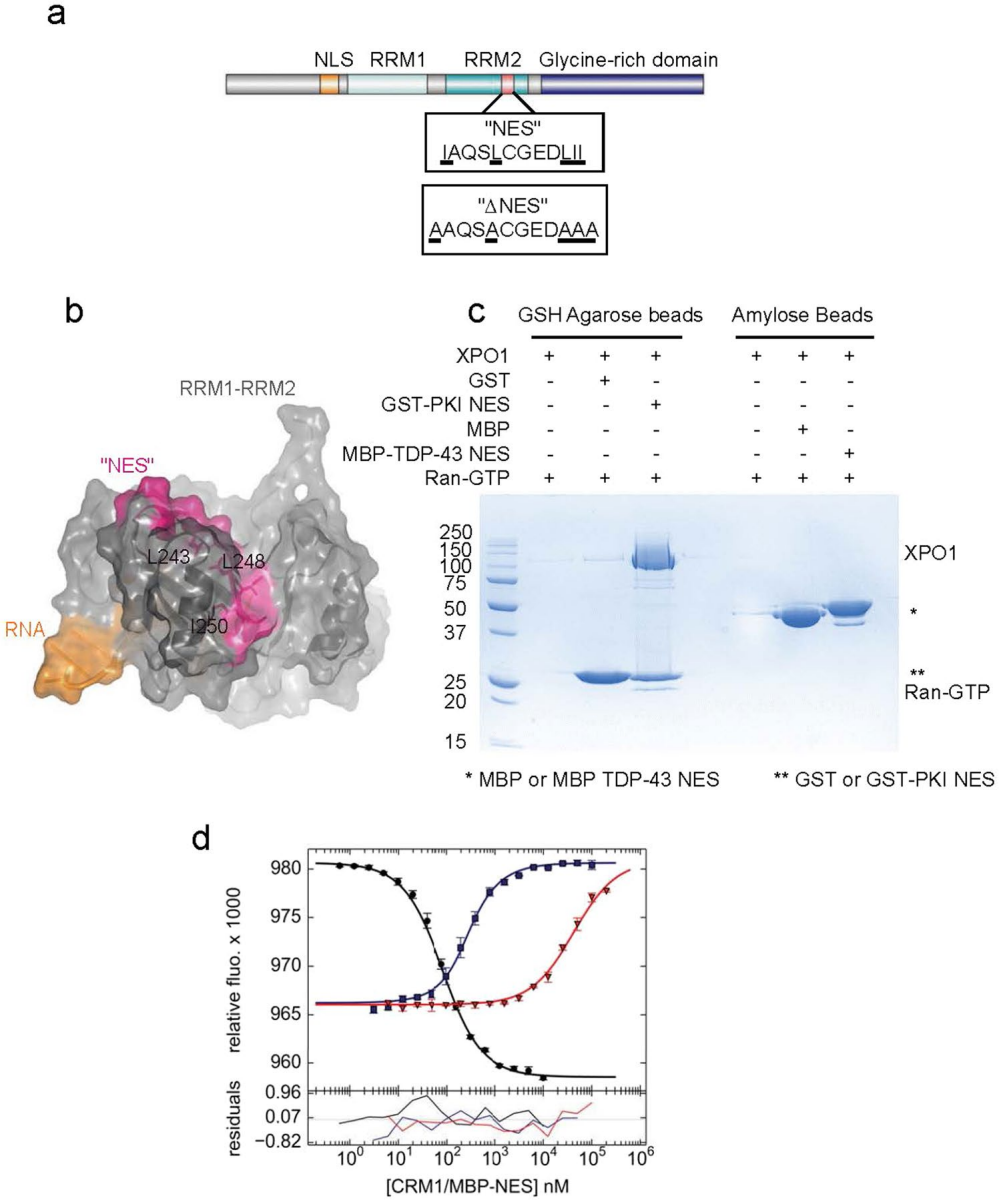
The putative NES in TDP-43 is not solvent exposed and has weak affinity for XPO1. (**a**) Domain organization of TDP-43, formatted using IBS Cuckoo^43^. NLS: Nuclear Localization Signal, residues 82–98. RRM1: RNA Recognition Motif 1, residues 106–165. RRM2: RNA Recognition Motif 2, residues 191–257. “NES”: putative Nuclear Export Signal, residues 239–250. “∆NES”: set of point mutations reported to disrupt putative NES, I239A, L243, L248A, I249A, I250A^18^. Glycine-Rich domain: residues 270–414. (**b**) NMR solution structure of the TDP RNA binding domain (RRM1-RRM2)^20^. Shown is the cartoon overlaid by a partly transparent solvent accessible surface. Residues comprising the putative NES have been colored magenta. The side chains of some critical hydrophobic residues – L243, L248, and I250 – are shown as sticks. (**c**) *In vitro* pull-down assay (Coomassie/SDS-PAGE) of purified human XPO1 binding to immobilized GST-NES_PKI_ (on GSH agarose beads) or MBP- NES_TDP_ (on amylose beads) in the presence of Ran-GTP (GSP1(179ter, Q71L)). (**d**) Competition differential bleaching assay. Binding of FITC-NES_PKI_ to XPO1 in the presence of excess RanGTP (GSP1 (179ter, Q71L)), measured by differential bleaching (blue line) Bleaching of a fluorescently labeled control NES, FITC-NES_PKI_, decreases with increasing XPO1 concentration (black line). MBP-NES_PKI_ competes with FITC-NES_PKI_ for XPO1 binding (blue line). MBP-NES_TDP-43_ poorly competes with FITC-NES_PKI_ for XPO1 binding (red line). Relative fluorescence of triplicate experiments are plotted on top with data points representing mean and standard deviation. Data fit residuals are plotted below.

Given the importance of TDP-43 localization in ALS, we sought to understand the determinants of normal TDP-43 trafficking. TDP-43 is predominantly nuclear, but constantly shuttling to and from the cytosol^17^. While TDP-43 mutations which affect its RNA binding, dimerization, and protein interactions have subtle effects on TDP-43 localization, the major determinants of TDP-43 localization are its nuclear import and nuclear export^17^. TDP-43 nuclear import is mediated by Importin-α, which binds to a canonical or classical K/R nuclear localization signal (cNLS) in the N-terminus of TDP-43 (Fig. 1a)^18,19^. Nuclear export of TDP-43 was proposed to be mediated by XPO1 (also known as CRM1), the exportin with the broadest cargo specificity, through direct binding to a classical nuclear export signal (NES)^18^. A putative NES was identified within the second of two tandem RNA Recognition Motifs (RRMs) which comprise the RNA-binding domain (Fig. 1a)^18^. However, there is little experimental evidence to support this export mechanism.

Here, we demonstrate that TDP-43 nuclear export is not mediated through the putative NES in RRM2. Our examination of the TDP-43 RRM1-RRM2 NMR structure (PDBID 4BS2)^20^ reveals that the reported NES is not solvent exposed, and therefore would not be accessible to bind XPO1. Moreover, XPO1 binding assays demonstrate that even the isolated putative NES peptide has very low affinity for XPO1. Finally, shuttling assays in cells demonstrate that RRM2 (which contains the putative NES) is not required for nuclear export of TDP-43. TDP-43 localization is further shown to be XPO1 independent, both in cultured HeLa cells and cultured primary hippocampal neurons. However, the fusion of a large (tdTomato) but not a small (flag) tag to TDP-43 is sufficient to significantly retard nuclear export. Together, these data support a model where TDP-43 nuclear export is largely diffusion mediated.

## Results

### The putative NES in RRM2 is not solvent exposed and the putative NES peptide does not tightly bind XPO1

XPO1 recognizes its cargos by directly binding a short peptide sequence within the cargo, termed a Nuclear Export Signal (NES). The NES binding groove in XPO1 accommodates diverse peptide sequences, so the NES consensus sequence is only loosely defined as regularly spaced hydrophobic residues^21,22^. It is therefore difficult to predict an NES based on sequence alone; predictions should take structural data into account, and must be experimentally verified.

The putative NES in TDP-43, “IAQSLCGEDLII” (residues 239–250, hydrophobic residues underlined) only poorly fits the consensus sequence, as there are no intervening non-hydrophobic residues between the last three hydrophobic residues^21^. Moreover, unlike most experimentally verified NESs which are located in unstructured or disordered regions of proteins, the putative NES in TDP-43 is located in a folded globular RNA Recognition Motif (RRM) domain (Fig. 1a)^18,23^.

As a rough guide to whether this proposed NES might bind XPO1, we examined a published NMR solution structure of the RNA-binding domain, RRM1-linker-RRM2 (PDBID 4BS2) (Fig. 1b)^20^. The residues comprising the putative NES (residues 239–250, colored magenta) contribute very little to the solvent accessible surface (Fig. 1b). Moreover, key hydrophobic residues within the putative NES – which normally directly contact the NES-binding groove of XPO1–are mostly buried in a surface representation of the RNA binding domain (Fig. 1b)^20,21^. To better visualize this, the side chains of L243, L248, and I250 are shown as sticks within the solvent accessible model of the RNA binding domain (Fig. 1b). These are clearly buried behind the proximal helix (Fig. 1b). In fact, calculating the solvent accessibility for the residues in the putative NES for TDP-43 predicts that all hydrophobic residues in this segment with the exception of Ile249 are predicted to be buried in the core of RRM1-RRM2 (Table 1). This suggests that these hydrophobic residues are likely critical for correct folding and function of the RNA-binding domain, but are not accessible for recruiting XPO1.

**Table 1:** Solvent accessibility surface of residues within TDP-43 putative NES.

Amino Acid	Residue #	Ratio (%)	In/Out
**ILE**	**239**	**16.5**	**i**
ALA	240	0	i
GLN	241	39.1	
SER	242	78.5	o
**LEU**	**243**	**6.3**	**i**
CYS	244	4.1	i
GLY	245	13.5	i
GLU	246	56.4	o
ASP	247	3.6	i
**LEU**	**248**	**7.8**	**i**
**ILE**	**249**	**25.5**	
**ILE**	**250**	**0.1**	**i**

Using the NMR data from Lukavsky *et al*.^20^ and an algorithm developed by Braun *et al*.^44^, the solvent accessibility of the putative NES was calculate (hydrophobic residues bolded). The first two columns are the amino acid identity and residue number. The third is the side chain surface area, normalized to a “random coil” of that residue. Resides are classified as solvent exposed if this normalized value exceeds 50% (labelled “o”) and buried if the normalized value is less than 20% (labelled “i”).

However, it is formally possible that these RRMs adopt alternative structures that might expose the putative NES and facilitate export. To test the ability of a fully-exposed putative NES peptide to bind XPO1, we expressed as a recombinant fusion protein in *E. Coli* the maltose binding protein fused to the putative NES peptide DDQ**IAQSLCGEDLII**KGI_236–253_ (MBP-NES_TDP_; putative NES in bold, hydrophobic residues underlined).

To qualitatively assess XPO1 binding, *in vitro* XPO1 pull-down assays were performed. MBP-TDP_NES_ was immobilized on amylose beads and incubated with XPO1 in the presence or absence of yeast RanGTP (GSP1). The TDP-43 putative NES was unable to pull down XPO1 in the presence of RanGTP, suggesting that even if RRM2 did adopt a conformation in which the putative NES were solvent exposed, XPO1 could not bind and mediate export. In contrast, the control NES GST-NES_PKI_, when immobilized on glutathione beads, was able to pull down XPO1 in the presence but not the absence of RanGTP (Fig. 1c).

Although XPO1 binding could not be detected by pulldown, we wanted to determine whether there was any weak binding. To quantitate the affinity of XPO1 for TDP-43 putative NES, we performed a competition differential bleaching experiment, as previously described^21^. Briefly, the binding affinity of MBP-NES_TDP_ for XPO1 was quantitated by its ability to compete with a labeled probe, FITC-NES_PKI_. The affinity of FITC-NES_PKI_ binding to XPO1 is determined by the kinetics of FITC bleaching, which is altered when the probe is bound to XPO1. The binding curve for the labeled FITC-NES_PKI_ probe yielded a dissociation constant (K_D_) of 73 nM, with 68.3% confidence interval [67, 79] (Fig. 1d, black circles). The competitive binding curve of MBP-NES_PKI_ with FITC-NES_PKI_ yielded similar result, K_D_ of 58 nM [47, 70], demonstrating the internal consistency of the assay (Fig. 1d, blue squares). The competitive binding curve of MBP-NES_TDP_ yielded a K_D_ of 13 μM for the interaction between XPO1 and the putative NES of TDP-43 [11.3, 15.4] (Fig. 1d, red triangles). A K_D_ of 13 µM is within the range of affinities expected of a weak NES^24^. However, the very weak affinity of the putative NES for XPO1, coupled with the structural evidence that it is not exposed, is very strong evidence that it is not directly binding XPO1 to mediate nuclear export of TDP-43.

### TDP-43 RRM2 does not contain an NES: TDP-43 RRM2 is not sufficient for nuclear export

Our results have shown that the putative NES “IAQSLCGEDLII” within RRM2 is not exposed to solvent and even when exposed in the form of a 16-residue peptide, binds XPO1 only very weakly. However, this does not exclude the possibility that RRM2 contains alternate nuclear export signals. To determine whether RRM2 contains trafficking signals, we assessed whether the RRM2 domain is sufficient and/or necessary for nuclear export of TDP-43.

**Figure 2:**
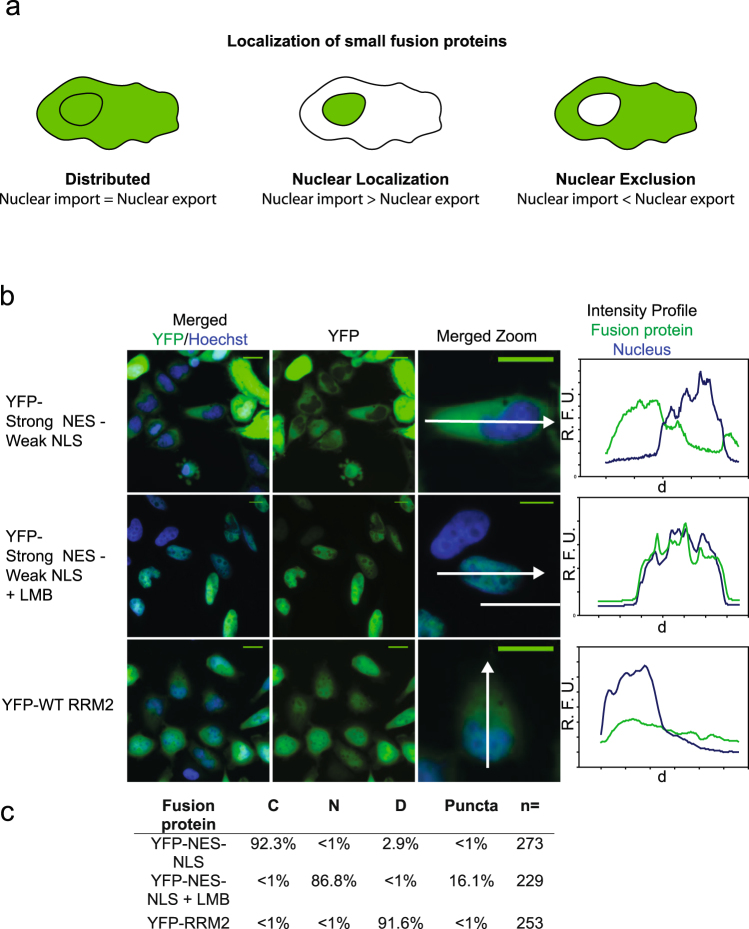
RRM2 is not sufficient for nuclear export. (**a**) Schematic of predicted localization of small fusion proteins. (**b**) Direct fluorescence of HeLa cells expressing the indicated fusion protein and treated with nuclear stain Hoechst. Merged image (Hoechst and YFP), YFP, and a close-up of Merged are shown. Scale bars are 10 um. Intensity plots for each image are shown; intensity was measured across the arrow in “Merged Zoom” panel. Intensity plots: y-axis is relative fluorescence, x-axis is distance. Indicated samples were treated with the XPO1 inhibitor Leptomycin B (LMB), 10 nM for 12 hours. (**c**) Cells from 3 independent experiments were categorized as nuclear “N”, “C” cytosolic, or “D” distributed. Apoptotic and mitotic cells were not categorized but were included in total cell count. Percentage of cells containing puncta is also indicated. n = number cells counted.

To determine whether RRM2 was sufficient for nuclear export, we fused the domain to a reporter, 2x eYFP. The small 2x eYFP-RRM2 fusion protein should be able to diffuse through the nuclear pore complex (NPC). If it contains no trafficking signals, it should be equally distributed between the cytosol and nucleus. However, if it contains trafficking signals, steady-state localization will depend on the relative strength of those signals; a stronger NLS will concentrate the reporter within the nucleus, while a stronger NES will concentrate the reporter in the cytoplasm (Fig. 2a). As a control for this assay, we assessed the localization of a fusion protein containing a strong NES and a weak NLS, 2x eYFP-NES_PKI_-NLS_SV40_, which is concentrated outside of the nucleus as predicted (Fig. 2b). However, when XPO1-mediated nuclear export is inhibited by treatment with the XPO1 inhibitor Leptomycin B, then the weak NLS prevails, and the 2x eYFP-NES_PKI_-NLS_SV40_ fusion protein accumulates within the nucleus. In contrast, the localization of 2x eYFP-RRM2 fusion protein is distributed throughout the cell, suggesting an absence of any export signals (Fig. 2b). To quantitatively assess the propensity for this localization, cells were counted and YFP signal was categorized as cytosolic, “C”, nuclear “N”, or distributed “D”. Cells which were either mitotic or apoptotic were not categorized, but were included in total cell counts (Fig. 2c).

To confirm the differential localization of these three YFP fusion proteins, intensity profiles were manually drawn for at least 20 cells for each condition. Using these intensity profiles, an average “nuclear” YFP signal, and an average “cytosolic” YFP signal were calculated for each cell. The ratio of these averages was taken as the metric of localization (Schema in Supplementary Fig. S1). Values for these three fusion proteins were significantly different, p < 0.001, when compared using the Mann-Whitney Rank sum test (Supplementary Fig. S1).

### TDP-43 RRM2 does not contain an NES: RRM2 is not required for nuclear export of TDP-43

**Figure 3:**
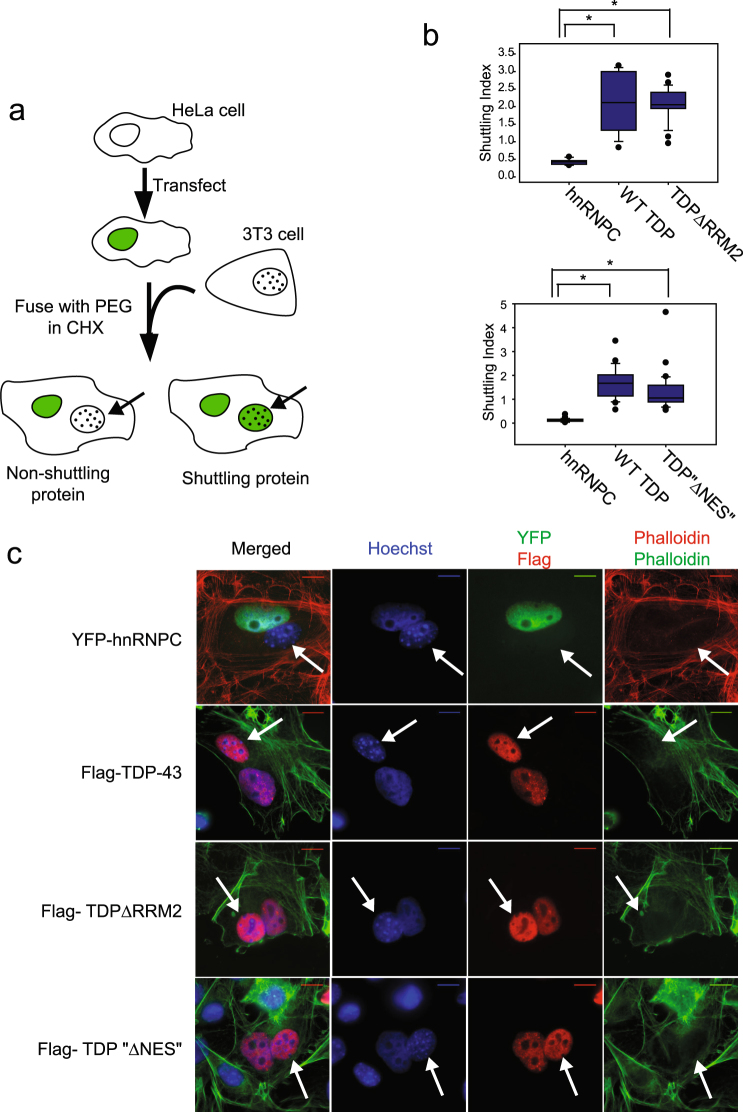
RRM2 is not required for nuclear export, and “∆NES” mutations do not abolish export. (**a**) Schematic of heterokaryon shuttling assay. (**b**) Quantification of shuttling assay. For each heterokaryon counted, the ratio of fluorescence in recipient nucleus/donor nucleus was plotted (normalized to background). Results are shown in box and whiskers plot. Top: 3 independent experiments. hnRNPC- 6 heterokaryons counted; WT TDP-43 −9 heterokaryons counted; TDP-43∆RRM2–19 heterokaryons counted. * indicates significant difference between groups, p < 0.002 using Mann-Whitney Rank Sum Test. No significant difference between WT and ∆RRM2. Quantification of shuttling assay from three independent experiments as above. hnRNPC- 22 heterokaryons counted; WT TDP-43–24 heterokaryons counted; TDP-43 “∆NES” – 26 heterokaryons counted. * indicates significant difference between groups, p < 0.001 using Mann-Whitney Rank Sum Test. No significant difference between WT and “∆NES”. (**c**) Images of sample heterokaryons shown. Nuclei are stained with Hoechst. YFP-hnRNPC is detected by direct fluorescence. Flag WT TDP-43, Flag TDP-43∆RRM2, and Flag TDP-43 “∆NES” are detected by immunofluorescence with Flag antibody. Actin cytoskeleton visualized with phalloidin stain. Recipient nucleus (3T3) indicated by arrow. Scale bars- 10 um.

To test whether RRM2 is required for TDP-43 nuclear export, the heterokaryon shuttling assay was used^25^. In these assays, a “donor” cell, which expresses a tagged protein of interest, is fused with a “recipient” cell to make a heterokaryon containing both nuclei. Cells are treated with the translation inhibitor, cycloheximide, to prevent newly synthesized cytosolic protein from accumulating in the recipient nucleus. Thus, accumulation of the tagged protein in the recipient nucleus indicates nuclear export from the donor nucleus and re-import into the recipient nucleus (Fig. 3a). The donor (HeLa) and recipient (3T3) nuclei can be distinguished by the distinctive speckling in 3T3 nuclei. To quantitate shuttling, fluorescence intensities in all nuclei of each heterokaryon were measured, and the ratio of recipient: donor nuclear fluorescence (termed “Shuttling index” or SI) was calculated. Sample images of analyzed heterokaryons are shown to illustrate the quantitation process, (Supplementary Fig. S2).

To confirm the integrity of the assay, YFP tagged hnRNPC was used as a negative control; hnRNPC, like TDP-43, belongs to the RNP family of proteins but does not shuttle^26^. As expected, YFP hnRNPC does not accumulate in the recipient nucleus (Fig. 3b) and fluorescence of the recipient nucleus is only ~5% of donor nucleus fluorescence, an SI of 0.05 (Fig. 3). In contrast, WT TDP-43, which was previously shown to shuttle, accumulates efficiently in the recipient nucleus with an SI >1 (Fig. 3)^17^.

If RRM2 contains the NES responsible for TDP-43 nuclear export, we would expect deletion of RRM2 to significantly decrease shuttling. However, TDP-43∆RRM2 accumulates in recipient nuclei to the same degree as wild-type TDP-43. Moreover, the “∆NES” point mutations, which were previously assumed to abolish nuclear export, do not affect TDP-43 shuttling; TDP-43”∆NES” accumulates in the recipient nucleus to the same degree as WT TDP-43 (Fig. 3b,c). Together, the evidence that RRM2 is neither sufficient for nuclear export, nor required for nuclear export of TDP-43, demonstrates that RRM2 does not contain an NES.

### “∆NES” mutations disrupt TDP-43 splicing function

The “∆NES” mutations ameliorate the toxicity associated with TDP-43 overexpression, both in *D melanogaster* eye and in cell culture systems^27,28^. This was hypothesized to be a result of inhibiting nuclear export of TDP-43. However, our data demonstrates that the “∆NES” mutations do not disrupt nuclear export of TDP-43 (Fig. 2b). Interestingly, the F147/9 L mutation, which disrupts TDP-43 splicing function, also ameliorates the toxicity of TDP-43 overexpression in *D melanogaster*^29^. We hypothesized that the “∆NES” mutations might also disrupt splicing function of TDP-43 because the hydrophobic residues in the (putative) “NES” reside in the hydrophobic core of the RNA binding domain.

To test the effect of the “∆NES” mutations on TDP-43 function, we assessed a known function of TDP-43; facilitating the splicing of CFTR exon 9. We performed *in vivo* splicing assays using the reporter minigene previously described^30^. This reporter was slightly modified; a silent mutation (1326A > G, K442 > K) was introduced in order to disrupt a cryptic splice site, decreasing formation of an intermediate splice product (also observed in the original report^30^). This point mutation did not interfere with the TDP-43 dependent exon skipping (Supplementary Figs S3, 4a).

**Figure 4:**
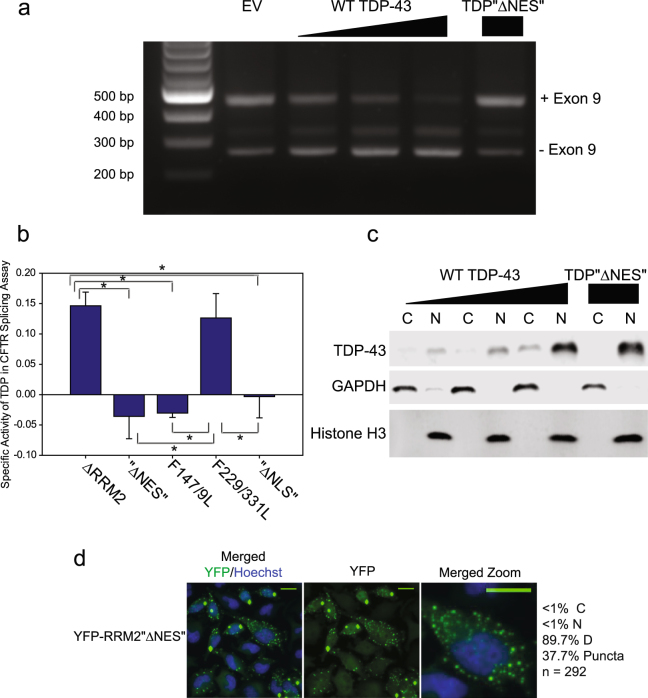
“∆NES” Mutations profoundly disrupt TDP-43 function. (**a**) CFTR exon splicing Assay. HeLa were cells co-transfected with the CFTR minigene and either empty vector (EV) or TDP-43. cDNA was PCR amplified and run on an agarose gel. Image is representative of three independent experiments. Uncropped image shown in Supplementary Fig. 4a. (**b**) qPCR analysis of HeLa cells co-transfected with the CFTR minigene and TDP-43. The “+9” and “−9” isoforms were detected with sequence specific primers. The specific activity: ((+9)/(−9))/(TDP-43) was calculated for each TDP-43 variant and normalized to WT (set to 1). The mean and standard deviation from three independent experiments are plotted (WT not shown). Statistical analysis using one-way ANOVA was performed, WT varied significantly from all mutants, with p < 0.001. * indicates additional significant difference between groups, p < 0.001. (**c**) Western blot of cytosolic “C”, and nuclear, “N”, fractions of lysate from cells in 3 A. GAPDH (Glyceraldehyde 3 phosphate dehydrogenase) acts as a marker and loading control for the cytosolic fraction, while Histone H3 acts as a marker and loading control for the nuclear fraction. Image is representative of three independent experiments. Uncropped images shown in Supplementary Fig. 4b. (**d**) Direct fluorescence of HeLa cells expressing the indicated fusion protein and treated with nuclear stain Hoechst. Merged image (Hoechst and YFP), YFP, and a close-up of Merged are shown. Scale bars are 10 um. Cells from 3 independent experiments were categorized as nuclear “N”, “C” cytosolic, or “D” distributed. Apoptotic and mitotic cells were not categorized but were included in total cell count. Percentage of cells containing puncta is also indicated. n = number cells counted.

As previously reported, WT TDP-43 mediated exon 9 skipping in a dose dependent manner (Fig. 4a,b, Supplementary Fig. S3). However, TDP-43 “∆NES” had no effect on exon 9 skipping, despite nuclear localization (Fig. 4a,b). To quantitatively assess the effect of TDP-43 mutations on CFTR exon 9 skipping, this assay was performed using qPCR. Splicing was assessed by calculating the ratio of (CFTR+9/ CFTR−9), using isoform specific primers. Splicing was normalized to TDP-43 expression to calculate a specific activity. Specific activities of all mutants were compared to WT TDP-43, which was set to 1. With this method, we compared the “∆NES” mutations with other mutations known to disrupt function: ∆NLS, which shifts TDP-43 distribution into the cytosol^18^; F147/9 L, RRM1 mutations which disrupt key RNA-stacking interactions^7^; and F229/331 L, RRM2 mutations analogous to F147/9 L (Fig. 4c)^7^.

Electrophoretic mobility shift assays have demonstrated that RRM1 is required for RNA-binding, while RRM2 is not^7^. Similarly, our CFTR splicing assays demonstrate that TDP-43 mutants with either RRM2 deletion or mutations (F229/331 L) still have residual function (~13% of WT), while an RRM1 mutation (F147/9 L) completely abrogates function (Fig. 4c). Interestingly, the “∆NES” point mutations do not spare residual function as do other RRM2 mutations. Lukavsky *et al*. demonstrated that one of the residues within the putative NES, Ile249, participates in a crucial RRM1-RRM2 interaction^20^. It may be that the “∆NES” mutations disrupt the geometry between RRM1, RRM2, and RNA, which is more disruptive than a simple RRM2 deletion.

Furthermore, introduction of the “∆NES” point mutations (Ile 239 Ala, Leu 243 Ala, Leu 248 Ala, Ile 249 Ala, Ile 250 Ala) in 2x eYFP-RRM2 causes the fusion protein to accumulate in puncta, suggesting these mutations disrupt its solubility (Fig. 4d). Indeed, these mutations were previously reported to disrupt the solubility of full-length TDP-43^18^. These results support the structural evidence that the residues within the putative NES are likely stabilizing the hydrophobic core of the RRM2 domain.

Interestingly, although 2x eYFP-RRM2 “∆NES” forms visible puncta in a substantial percentage of transfected cells (~38%, Fig. 4d), we do not observe puncta in cells expressing TDP-43 “∆NES”. However, other groups have observed that TDP-43 ∆NES forms nuclear puncta; this may depend on the level of overexpression^18^.

### Distribution of the TDP trafficking mutant Flag TDP∆NLS is unaffected by LMB treatment

Our work has invalidated claims that the putative NES within RRM2 is mediating nuclear export of TDP-43. However, we are still left with the question of how TDP-43 nuclear export is mediated. The most likely candidate is XPO1, the exportin with the broadest cargo specificity. To determine whether TDP-43 nuclear export is dependent on XPO1, we first assessed endogenous TDP-43 localization in the presence or absence of XPO1 siRNA (Supplementary Fig. S4). However, endogenous TDP-43 is almost exclusively nuclear and cytosolic TDP-43 levels are too low to visualize. XPO1 knockdown, which was confirmed by western blot, had no apparent effect on TDP-43 localization, but the indistinguishable cytosolic TDP-43 levels in untreated cells made this result impossible to interpret (Supplementary Fig. S4). We also assessed the effect of the XPO1 inhibitor LMB on Flag WT TDP-43 localization^31^. Again, the extremely low cytosolic TDP-43 levels prevented a determination of whether LMB treatment diminished cytosolic TDP-43 (Supplementary Fig. S4).

**Figure 5:**
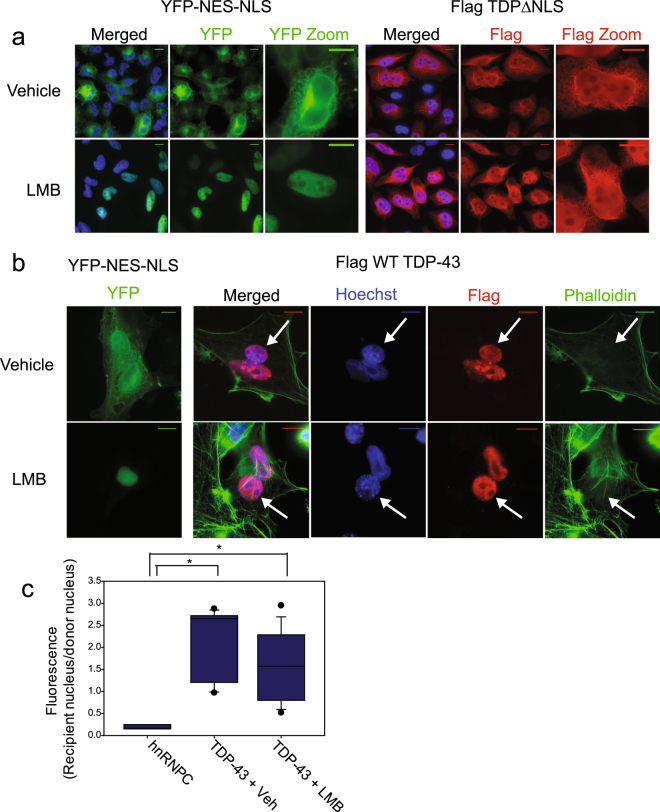
TDP-43 localization and nuclear export is XPO1 independent. (**a**) Direct fluorescence of HeLa cells expressing fusion protein YFP-NES_PKI_-NLS_SV40_ on left. Immunofluorescence using Flag antibody on HeLa Tet-ON cells expressing Flag TDP-43∆NLS on the right. Nuclei were stained using Hoechst. Cells were treated with either Vehicle (Ethanol, −0.1% of total volume) or Leptomycin B (LMB) 10 nM for 12 hours. (**b**) Left: direct fluorescence of HeLa cells undergoing mock shuttling assay, expressing YFP-NES_PKI_-NLS_SV40_. Cells were treated with either Vehicle (Ethanol, −0.1% of total volume) or Leptomycin B (10 nM) for the duration of the assay. Right: Example heterokaryons from shuttling assay. Cells were treated with either Vehicle (Ethanol, −0.1% of total volume) or Leptomycin B (10 nM) for the duration of the assay. Nuclei are stained with Hoechst. Flag WT TDP-43 is detected by immunofluorescence with Flag antibody. Actin cytoskeleton visualized with phalloidin stain. Recipient nucleus (3T3) indicated by arrow. Scale bars- 10 um. (**c**) Quantification of shuttling assay as in 1b. Two independent experiments. hnRNPC- 3 heterokaryons counted. Flag WT TDP-43, vehicle treated- 8 heterokaryons counted. Leptomycin B treated- 10 heterokaryons counted. * indicates significant difference between groups, p < 0.002 using Mann-Whitney Rank Sum Test. No significant difference between Vehicle and LMB treated heterokaryons, but insufficient power to detect a difference.

To visualize cytosolic TDP-43, we expressed Flag-tagged TDP∆NLS, which contains mutations that disrupt its cNLS^18^. As previously reported in the literature, the TDP∆NLS mutant is not excluded from the nucleus^17^ (Fig. 5a). Therefore, inhibition of TDP-43 nuclear export should result in an accumulation of TDP-43 ∆NLS in the nucleus, and a visible depletion of cytosolic TDP∆NLS. Treatment with LMB had no effect on cytosolic TDP∆NLS, suggesting that TDP-43 nuclear export is XPO1 independent (Fig. 5a). XPO1 inhibition was efficient; a 2x eYFP-NES-NLS reporter construct was nuclear in LMB treated cells (Fig. 5a).

### Shuttling of TDP-43 is unaffected by Leptomycin B treatment

To more directly test the effect of XPO1 inhibition on TDP-43 nuclear export, heterokaryon shuttling assays were performed in the presence or absence of the XPO1 inhibitor Leptomycin B. Shuttling of TDP-43 was unaffected by Leptomycin B (Fig. 5b,c). Again, XPO1 inhibition was assessed by localization of the YFP-NES_PKI_-NLS_SV40_ reporter, which was cytosolic in the vehicle treated cells and nuclear in LMB treated cells (Fig. 5b). YFP-hnRNPC was also used as a negative control for shuttling (Fig. 5c).

As an additional control, we constructed a Leptomycin B sensitive TDP-43 fusion protein: NES_REV_TDP-43 (TDP-43 fused to an XPO1-dependent NES from the HIV protein Rev) (Supplementary Fig. S4). As expected, this fusion protein accumulated in the nucleus after Leptomycin B treatment, further supporting the assertion that TDP-43 does not contain an XPO1 dependent NES (Supplementary Fig. S4).

### Distribution of TDP-43 in rat hippocampal neurons is both cytosolic and nuclear and unaffected by LMB treatment

While our results demonstrate that TDP-43 localization is XPO1 independent in HeLa cells, it is possible that the mechanism of TDP-43 nuclear export might vary based on cell type. Therefore, it was important to confirm the XPO1 independence in the most relevant cell type, neurons.

**Figure 6:**
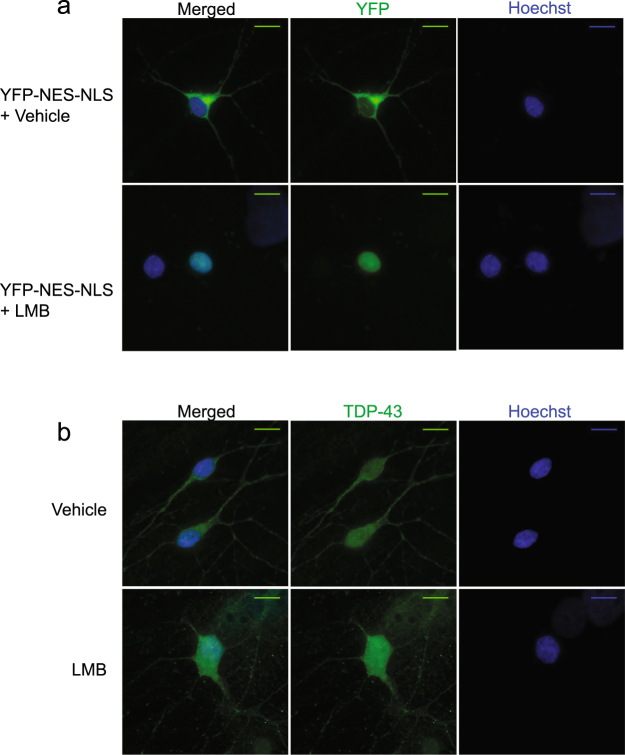
TDP-43 localization in cultured neurons is XPO1 independent. (**a**) Hippocampal neurons were isolated and cultured. Indicated neurons were transfected with the reporter YFP-NES_PKI_-NLS_SV40_ and treated with either vehicle (Ethanol, 0.1% of total volume) or Leptomycin B (LMB) 10 nM for 7 hours. Nuclei were stained with Hoechst and YFP-NES_PKI_-NLS_SV40_ was detected with direct fluorescence. Images are representative of three independent experiments. Scale bar- 10 um. (**b**) Indicated neurons were treated with either vehicle (Ethanol, 0.1% of total volume) or Leptomycin B (LMB) 10 nM for 7 hours. Nuclei were stained with Hoechst and endogenous TDP-43 was detected with immunofluorescence using a TDP-43 antibody. Images are representative of three independent experiments. Scale bar- 10 um.

To determine the correct dose of LMB to inhibit XPO1 in cultured hippocampal neurons, we transfected neurons with the reporter YFP-NES_PKI_-NLS_SV40_. While transfection efficiency was low, the reporter was highly expressed in transfected cells, and was excluded from the nucleus as predicted (Fig. 6a). LMB treatment of 10 nM for 7 hours was sufficient to redistribute the reporter from the cytosol to the nucleus (Fig. 6a).

Next, we repeated these conditions and assessed endogenous TDP-43 localization using immunofluorescence (Fig. 6b). Unlike in HeLa cells and *in situ* neurons, TDP-43 is evenly distributed between the cytosol and nucleus in cultured neurons. Therefore, inhibiting TDP-43 nuclear export should lead to a profound change in localization. However, LMB treatment had no effect on TDP-43 localization, suggesting that TDP-43 localization is XPO1 independent in neurons (Fig. 6b).

### tdTomato tag disrupts passive but not active nuclear import of TDP-43

**Figure 7:**
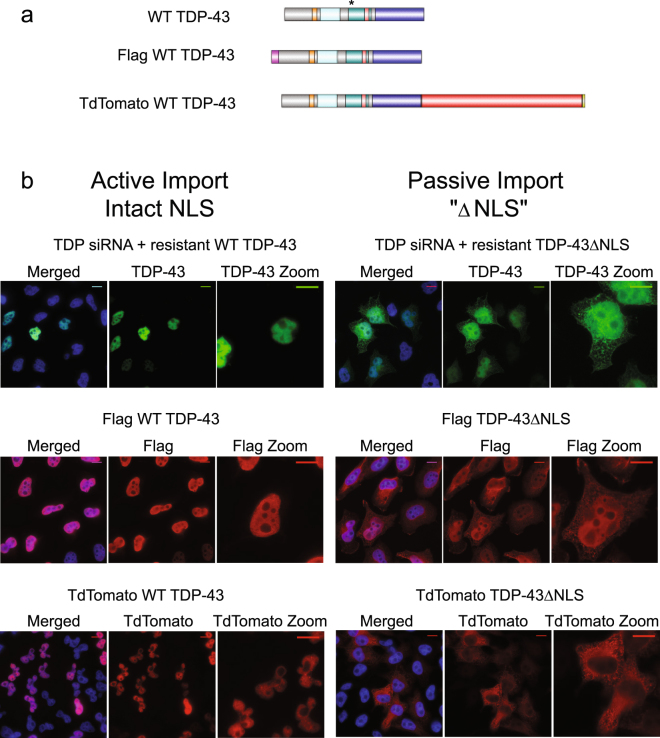
Addition of a bulky tag on TDP-43 disrupts passive but not active nuclear import. (**a**) Domains maps (to scale) of WT TDP-43 containing synonymous mutations which confer resistance to TDP-43 siRNA (indicated by asterisk), Flag WT TDP-43, tdTomato TDP-43. Formatted using IBS cuckoo^43^. (**b**) Immunofluorescence of HeLa cells expressing the indicated protein. Selected samples were treated with TDP-43 siRNA to deplete endogenous TDP-43. Resistant TDP-43 was visualized via immunofluorescence with TDP-43 antibody; Flag TDP-43 was visualized via immunofluorescence with Flag antibody; tdTomato TDP-43 was visualized with direct fluorescence. Nuclei were visualized using Hoechst stain. Scale bar- 10 um. Images are representative of 3 independent experiments.

We had previously noted that the FlagTDP-43∆NLS mutant is not excluded from the nucleus. To confirm that this is a property of TDP-43∆NLS, and not an artifact introduced by the Flag tag, we made synonymous mutations in both WT TDP-43 and TDP-43∆NLS to confer resistance to an individual TDP-43 siRNA. Efficient knockdown of endogenous TDP-43 and addback of resistant WT TDP-43 and resistant TDP-43∆NLS was confirmed with immunofluorescence and western blotting (Fig. 7b, Supplementary Fig. S5). Addback experiments confirmed that, like Flag TDP-43∆NLS, untagged TDP-43∆NLS is partially nuclear (Fig. 7b).

There are two possible explanations for this: first, it is possible that TDP-43 is small enough to passively diffuse through the nuclear pore. This would be consistent with some of the recent literature^32^. Second, it is conceivable that TDP-43 contains a redundant NLS elsewhere. To distinguish between these possibilities, we assessed the localization of a ~93 kDa tdTomato-TDP-43 fusion protein, which is predicted to be too large to efficiently diffuse through the nuclear pore. We then mutated key residues within the bipartite NLS (K82A, R83A, and K84A)^18^, and termed the mutant “tdTomato TDP-43∆NLS”. Interestingly, this mutant was almost entirely excluded from the nucleus (Fig. 7b).

We also set out to formally exclude that possibility that TDP-43 contained an alternate NLS, which was somehow occluded by the tdTomato tag (which is C-terminal toTDP-43, while the flag tag is N-terminal to TDP-43). To do this, we created several additional fusion proteins with various regions of the C-terminus (residues 271–414) fused to the reporter YFP. If any region of the C-terminus contained an NLS, the reporter should be nuclear. However, all fusion proteins were distributed between the cytosol and nucleus, suggesting the C-terminus does not contain an NLS (Supplementary Fig. S5). As before, the propensity of distribution of each YFP fusion protein was also quantified (Supplementary Fig. 7c).

Together, these results demonstrate that TDP-43 is compact enough to diffuse through the NPC.

### tdTomatoTDP-43 nuclear export is impaired relative to Flag TDP-43 nuclear export

**Figure 8:**
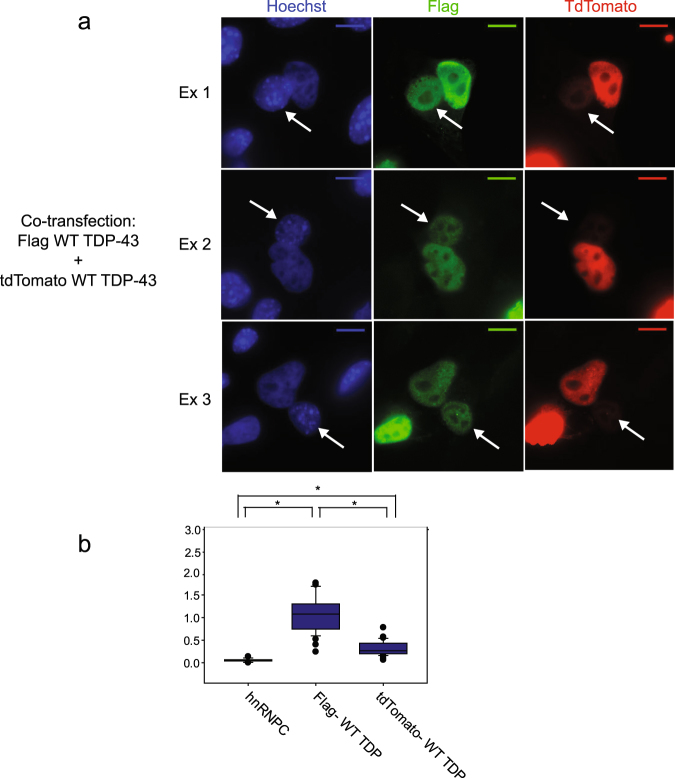
Addition of a bulky tag on TDP-43 disrupts nuclear export. (**a**) Example heterokaryons from a variation of the heterokaryon shuttling assay. HeLa cells were co-transfected with Flag TDP-43 and tdTomato TDP-43 and shuttling assay was performed as in Fig. 3. Heterokaryons were identified by presence of Flag TDP-43 within a recipient nucleus. Flag TDP-43 was visualized via immunofluorescence with Flag antibody, tdTomato TDP-43 was visualized via direct fluorescence. Nuclei were detected using Hoechst stain. Images are representative of 4 independent experiments. Recipient nuclei are indicated by arrow. Scale bar- 10um. (**b**) Quantification of shuttling assay as in 1b. Results are pooled from 4 independent experiments. hnRNPC- 15 heterokaryons counted. Cotransfected with Flag TDP-43 and tdTomato TDP-43–28 heterokaryons counted. * indicates significant difference between groups, p < 0.001. To compare hnRNPC vs Flag TDP-43, and hnRNPC vs tdTomato TDP-43, Mann Whitney rank sum test was used. To compare Flag TDP-43 vs tdTomato TDP-43, Wilcoxon signed rank test was used.

Unlike active nuclear import and export, passive diffusion through the nucleus is intrinsically bidirectional. Therefore, the observation that TDP-43 passively diffuses into the nucleus implies that TDP-43 also passively diffuses out of the nucleus. We wanted to determine the contribution of passive diffusion to nuclear export of TDP-43. To do this, shuttling of Flag TDP-43 (which can passively diffuse through the nuclear pore) was directly compared to shuttling of tdTomato TDP-43 (which diffuses much less efficiently). Heterokaryon assays were performed in which donor cells co-expressed Flag TDP-43 and tdTomato TDP-43. Shuttling index for Flag TDP-43 and tdTomatoTDP-43 was calculated as above (Fig. 8b). As seen earlier, Flag TDP-43 efficiently accumulates in the recipient cell, with a shuttling index >1. However, tdTomato TDP-43 accumulation in the recipient nucleus is significantly lower, with a shuttling index of ~0.3. We used the paired Wilcoxon signed rank test to compare the shuttling index of Flag TDP-43 to tdTomato TDP-43 for each heterokaryon. The shuttling of tdTomato WT TDP-43 was significantly impaired compared to Flag WT TDP-43 (p < 0.001).

Again, we wanted to formally exclude the possibility that the C-terminus contained an NES, which was occluded by the large N-terminal tdTomato tag. To do this, we assessed the contribution of the C-terminus to export. Heterokaryon shuttling assays with Flag TDP-43 ∆C-terminus (TDP-43 residues 1-269) demonstrated that the C-terminus is not required for shuttling (Supplementary Fig. S6). Together, these data suggest that the primary effect of the tdTomato tag on shuttling is size dependent. As passive export is strongly affected by size, this suggests that the primary driver of TDP-43 nuclear export is diffusion^32^.

## Discussion

TDP-43 mislocalization plays a causal role in the toxicity of ALS, but the cellular insults which lead to mislocalization are largely unknown. We sought to identify determinants of normal TDP-43 trafficking in order to better understand and possibly disrupt the forces that lead to TDP-43 mislocalization in ALS. Here, we have shown that TDP-43 nuclear export is not mediated by the putative XPO1 dependent NES previously reported. While we did not assess other potential routes of nuclear export, others have demonstrated that TDP-43 nuclear export does not require either the exportin XPO5 or the mRNA export factors Aly/REF^33^.

The available evidence strongly suggests that TDP-43 nuclear export is a result of passive diffusion. First, we showed that untagged TDP-43, as well as TDP-43 tagged with the small Flag protein, diffuses through the NPC. As expected, this diffusion is size-dependent, and is markedly reduced if TDP-43 is fused to the large tag tdTomato. Second, we demonstrate that nuclear export of tdTomatoTDP-43 – which cannot efficiently diffuse through the NPC—is significantly impaired relative to Flag TDP-43. Finally, another group found that nuclear export of an enlarged TDP-43 fusion protein is highly inefficient^33^. Thus, two different large tags (tdTomato, 54 kDa and GR_2_-GFP_2_, 119 kDa), fused to different termini of TDP-43 (C-terminus and N-terminus) had the same effect: inhibition of nuclear export. Two different small tags (V5, ~1 kDa, and 3xFlag, ~3 kDa) fused to TDP-43 did not inhibit nuclear export. This strongly supports the notion that TDP-43 nuclear export is size dependent, and therefore predominantly diffusion limited.

This has broad implications for the therapeutic strategies which might be used to correct TDP-43 mislocalization. Because TDP-43 nuclear export is driven by diffusion rather than requiring active transport, targeting nuclear export with a small molecule inhibitor is not feasible. Moreover, it suggests that XPO1 inhibitors, which have been successful in preclinical models of several cancers and multiple sclerosis, would not correct TDP-43 mislocalization in ALS^34–37^. However, XPO1 inhibitors may still ameliorate the course of ALS, by modifying the localization of downstream mediators of toxicity.

This finding also raises questions about the role of TDP-43 in the cytosol. Some roles for TDP-43 in the cytosol have been identified, such as axonal transport of one of its target mRNAs, association with translation and splicing machinery, and recruitment into stress granules^38,39^. However, it has also been recognized that TDP-43 is prone to cytosolic aggregation, in a concentration dependent manner. Together, these would predict very tight regulation of cytosolic TDP-43 levels. This is not consistent with the unregulated, diffusion driven nuclear export of TDP-43 that we have observed. It is possible that cytosolic TDP-43 levels are in fact controlled by nuclear import and retention alone. It is also conceivable that the roles of TDP-43 in the cytosol, which are not well-characterized, are not as general or as critical for cellular function as has been proposed.

Finally, the finding that this putative NES within TDP-43 RRM2 is not a true export signal raises questions about other putative NESs within RRMs, which have been predicted in several other members of the RNP family of proteins: FUS, TAF15, EWSR1, hnRNPA1, hnRNPA2B1^40^. For the most part, these predicted NESs have not been verified experimentally, and it remains to be seen whether they are truly mediating export. In fact, recent evidence suggests that at least one of these, the FUS NES, is also a false NES^33^. Possibly, nuclear export via passive diffusion is conserved throughout this family of shuttling proteins.

## Materials and Methods

Constructs—cDNA encoding human TDP-43 (accession number NM 007375) in the plasmid pBUDCE was a kind gift from Jeffrey Elliot. tdTomato-TDP-43 was purchased from Addgene (Catalog # 28205). The YFP-shuttle reporter, eYFPx2-PKI NES-SV40 NLS fusion protein, was a gift from YuhMin Chook. CFTR T5 minigene was a gift from Francisco Baralle. Human hnRNPC cDNA was purchased as a Ultimate ORF clone (ThermoFisher Scientific Clone ID IOH7506). Vectors were modified as follows. Flag-TDP-43 contains an N-terminal Flag tag. The T5 minigene was modified with the synonymous mutation 1326 A >G in an alternate splice acceptor site to reduce an intermediate splicing product (Supplementary Fig. S2). GFP-hnRNPC contains a C-terminal GFP tag. Fusion proteins and deletions were generated using overlap extension PCR. Point mutations were generated using site-directed mutagenesis. All cloning was confirmed by sequencing.

siRNA resistant TDP-43 constructs contained the following synonymous mutations: 525C > T, 531 T > A, 534 T > A, 537T > C

With the exception of tdTomato-TDP-43, all TDP ∆NLS mutants contain the mutations also referred to as “∆NLS1/2”: K82A, R83A, K84A; K95A, K97A, R98A.

tdTomato-TDP-43∆NLS contains only a subset of these mutations:“∆NLS 1”: K82A, R83A, K84A.

Rev NES TDP-43: contains an N-terminal NES from the HIV Rev protein “LPPLERLTL”.

Flag“ ∆C-term”: N terminal Flag Tag, TDP-43 residues 1–269

Flag TDP ∆RRM2: N terminal Flag tag, TDP del 191–257

YFP-RRM2: eYFPx2 fused to TDP-43 residues, 185–269

YFP- C terminal constructs: eYFPx2 fused to TDP-43 residues 271–345, 311–380, or 346–414.

### Protein expression and purification

The TDP-43 putative NES with 3 surrounding residues, was cloned into pMAL-TEV. PKI-NES and TDP-43 “NES” were expressed in BL21 E. Coli, and induced at 25 degrees celsius with 0.5 mM isopropyl β-D-1-thiogalactopyranoside for 10 hours. Cells were lysed in lysis buffer (500 mM Tris pH 7.5, 200 mM NaCl, 10% glycerol, 2 mM DTT, leupeptin, benzamide, pefablock). Then PKI-NES and TDP “NES” were purified using affinity chromotography with amylose beads and then ion exchange chromotography (HiTrap Q, GE Healthcare Life Sciences) using a 0 to 15% NaCl gradient. Purified MBP-NESs were concentrated and buffer exchanged overnight for use in downstream assays.

Ran-GTP (GSP1 179ter, Q71L) and XPO1 (CRM1) were expressed and purified as previously described^21^.

### Pull down binding assays and competition bleaching experiments

Pull down binding assays and competition bleaching experiments were performed as described in Fung *et al*.^21^. Data for competition bleaching experiments was analyzed in PALMIST, and binding curves were generated using GUSSI.

### Cell Culture

HeLa Tet-ON (referred to within the manuscript as HeLa) cells were used for ease of maintenance and transfection. HeLa Tet-ON (Clontech, Mountain View, CA) cells and 3T3 cells were maintained in Dulbecco’s modified Eagle’s medium supplemented with 10% fetal bovine serum, 1% penicillin/streptomycin at 37 degrees in bank percentage oxygen. Hippocampal neurons were isolated from C57/BL6 rat P2 pups and cultured as previously described in Li *et al*.^41^. All animal procedures conformed to the guide for the care and use of laboratory animals and were approved by the Institutional Animal Care and Use Committee at UT Southwestern Medical Center.

### Knockdown, transfection, Drugs, and antibodies

siRNAs targeting TDP-43 and XPO1 were synthesized by Dharmacon. For TDP-43, an individual siRNA was used (D-012394-04, seq:GCAAACUUCCUAAUUCUAA). For XPO1, a pool was used: M-003030-02-0005, seq: GAAAGUCUCUGUCAAAAUA, GCAAUAGGCUCCAUUAGUG, GGAACAUGAUCAACUUAUA, GGAUACAGAUUCCAUAAAU.

Cells were transfected with siRNAs using Lipofectamine RNAiMax (ThermoFisher 13778150) according to manufacturer’s instructions. All transfections (including hippocampal neurons) were performed using Lipofectamine 2000 (ThermoFisher 11668019) according to manufacturer’s instructions. Leptomycin B was purchased from LC Laboratories (Cat # L-6100), and stored as an ethanol solution at −20. HeLa Tet-ON cells were treated with 10 nM for 12 hours, and hippocampal neurons with 10 nM for 7 hours.

Primary Antibodies: GAPDH (Cell Signaling 2118), TDP-43 (Proteintech 10782-2-AP), Histone H3 (Abcam ab1791), Flag (Sigma M2, F1804), XPO1 (Santa Cruz sc-5595).

Secondary Antibodies (for western blotting): IRDye 680RD Goat anti-Rabbit (LI-COR 926–68071), IRDye 800CW Goat anti-Mouse (LI-COR 926–32210).

Secondary Antibodies (for immunofluorescence): Alexa Fluor 488 Goat anti-mouse (Life Technologies A11001), Alexa Fluor 488 Goat anti-rabbit (Life Technologies A11008), Cy3 Goat anti-mouse (Jackson Immunoresearch 115-165-003).

### CFTR Splicing Assay

HeLa Tet-ON cells were co-transfected with T5 CFTR minigene (containing blank mutation) and either pBUD TDP-43 or empty vector. 24 hours later, RNA was harvested using the Nucleospin RNA kit (Macherey-Nagel 740955.50). cDNA was synthesized using high-capacity cDNA reverse transcription kit (Applied Biosystems 4368814). Splicing was assessed either a) by PCR amplification of CFTR exon 9 using flanking primers (see table) followed by gel electrophoresis to separate splice variants, detected with ethidium bromide or b) quantitative real-time PCR with splice specific primers (see Table).

### Quantitative real-time PCR and calculation of TDP-43 specific activity

Quantitative PCR was performed on blank machine using Power SYBR Green PCR Master Mix (Applied Biosystems 4367659). HPRT was used as a loading control, and relative transcript levels were calculated using the ∆∆ Ct method.

To calculate TDP-43 specific activity, each experiment contained cells co-transfected with empty vector and three concentrations of WT TDP-43 (0.05 ug, 0.15 ug, and 0.5 ug). Cells expressing all other TDP-43 variants were transfected with one concentration, 0.5 ug. Transcript levels of TDP-43, CFTR +9, and CFTR −9 were calculated using quantitative real-time PCR, using the delta delta Ct method with HPRT as a loading control. For each sample, normalized splicing index was calculated as: Normalized splicing index = (CFTR +9)/((CFTR −9)(TDP-43)). The normalized splicing index for the three WT samples was averaged. The specific activity of each TDP-43 mutant was calculated as: specific activity of mutant = (normalized splicing index of mutant)/(average normalized splicing index of WT). For each mutant, the specific activity is the average of at least three independent experiments.

### Nuclear and cytosolic fractionation

Fractionation was performed as described in Gagnon *et al*.^42^. Briefly, cells were counted and then lysed in a proportional volume of hypotonic lysis buffer (10 mM Tris, pH 7.4, 10 mM NaCl, 3 mM MgCl_2_, 0.3% NP-40, 10% glycerol, Protease inhibitor tablet and NaVO_4_) with light agitation. After a low speed spin cytosolic fraction was removed, and NaCl was added for a final concentration of 150 mM NaCl. Intact nuclei were rinsed once more with hypotonic lysis buffer, then resuspended in nuclear lysis buffer (20 mM Tris, pH 7.4, 150 mM NaCl, 3 mM MgCl_2_, 0.3% NP-40, 10% glycerol). Cytosolic and nuclear lysates were boiled at 100 degrees celsius for 5 min in 1x Laemmli buffer, then run on a 10% Tris-Gly gel and transferred onto a PVDF membrane (Millipore). Primary and secondary antibodies used for detection listed above.

Blot was scanned using an Odessey cLx.

### Live cell imaging and quantification of localization of YFP fusion proteins

HeLa Tet-ON cells were plated in 24 well dishes and transfected as usual 24 hour later. Cells were incubated overnight, then nuclei were stained with Hoescht (Invitrogen 33342), and nuclei and fusion proteins were detected with direct fluorescence. Images are from 20X or 40X objective, gathered using Nikon Eclipse TE2000-U microscope and Photometrics CoolSNAP ES2 camera. Path analysis on >20 representative cells from 2 independent experiments was performed using the Nikon elements Software, and values were exported to Excel. The cytosol was defined as a Hoechst signal <50 R.F.U.s, and the nucleus as a Hoechst signal >500 R.F.U.s. The average cytosolic YFP and the average nuclear YFP signal were calculated. The ratio Nuclear YFP/Cytosolic YFP was used as a metric of localization.

### Statistical Analysis

Statistical analysis was performed using SigmaPlot 12.0 software. Statistical tests, sample size, and p-value are indicated in legends.

### Immunofluorescence

HeLa Tet-ON cells or hippocampal neurons were plated on glass coverslips and treated as indicated. Cells were processed for immunofluorescence as follows: 10 minute fixation in 4% Paraformaldehyde, 10 minute permeabilization in 0.5% Triton-X 100, 30 minute blocking in 10% Normal Goat Serum (Invitrogen 50062Z). Cells were incubated with primary antibodies overnight (listed above) and secondary antibodies for 1 hour at room temperature, followed by Hoechst staining (Invitrogen 33342) and/or phalloidin staining(Alexa Fluor 555 Phalloidin, Life Technologies A34055). Coverslips were mounted on glass slides using Prolong Gold antifade mounting reagent (Life Technologies P36934) and visualized using the 60X objective on the Nikon Eclipse TE2000-U. Images were obtained using Photometrics CoolSNAP ES2 camera. Images were analyzed using Nikon Elements software.

### Heterokaryon shuttling assay and calculation of shuttling index

HeLa-Tet-ON cells were transfected, and 24 hours later were co-plated with 3T3 cells on glass coverslips. Cells were incubated for 3 hours, then cycloheximide was added (100 ug/ml) and cells were incubated an additional 30 minutes. Then slides were inverted on 50% poly ethylene glycol in serum-free media for 2 minutes to form heterokaryons. Slides were rinsed in PBS and returned to cycloheximide-containing media. Cells were incubated an additional 3 hours then processed for immunofluorescence as usual.

Heterokaryons were confirmed by visualizing the actin cytoskeleton. At least 5 heterokaryons were counted for each condition during each experiment, with at least three independent experiments.

Nuclear fluorescence was quantified by manually outlining the nuclei (Hoechst stained) and comparing to a manually outlined control using NIS elements software. Shuttling index, or “SI” was calculated as the normalized ratio of recipient nucleus fluorescence/donor nucleus fluorescence: (3T3 fluorescence - background)/(HeLa Tet-ON nuclear fluorescence- background).

### Data Availability

No datasets were generated or analyzed during the current study.

## Electronic supplementary material

Supplementary Figures
