# Supplementary material for: Active nuclear import and passive nuclear export are the primary determinants of TDP-43 localization
Source: Sci Rep. 2018 May 4;8:7083. doi: 10.1038/s41598-018-25008-4 (PMC5935693; doi:10.1038/s41598-018-25008-4)
Supplement: Supplementary file 1 — Supplementary Figures [file 41598_2018_25008_MOESM1_ESM.pdf]

Active nuclear import and passive nuclear export are the primary determinants of TDP-43 localization

Authors: Emile S. Pinarbasi[1,2], Tolga Cağatay[3], Ho Yee Joyce Fung[3], Ying C Li[2, 4], Yuh Min Chook[3], Philip J. Thomas[1*]

[1]Department of Physiology, UT Southwestern Medical Center at Dallas, Dallas, TX 75390

[2]Medical Scientist Training Program, UT Southwestern Medical Center at Dallas, Dallas, TX, 75390

[3]Department of Pharmacology, UT Southwestern Medical Center at Dallas, Dallas, TX 75390

[4]Department of Neuroscience, UT Southwestern Medical Center at Dallas, Dallas, TX 75390

Supplementary Figure S1

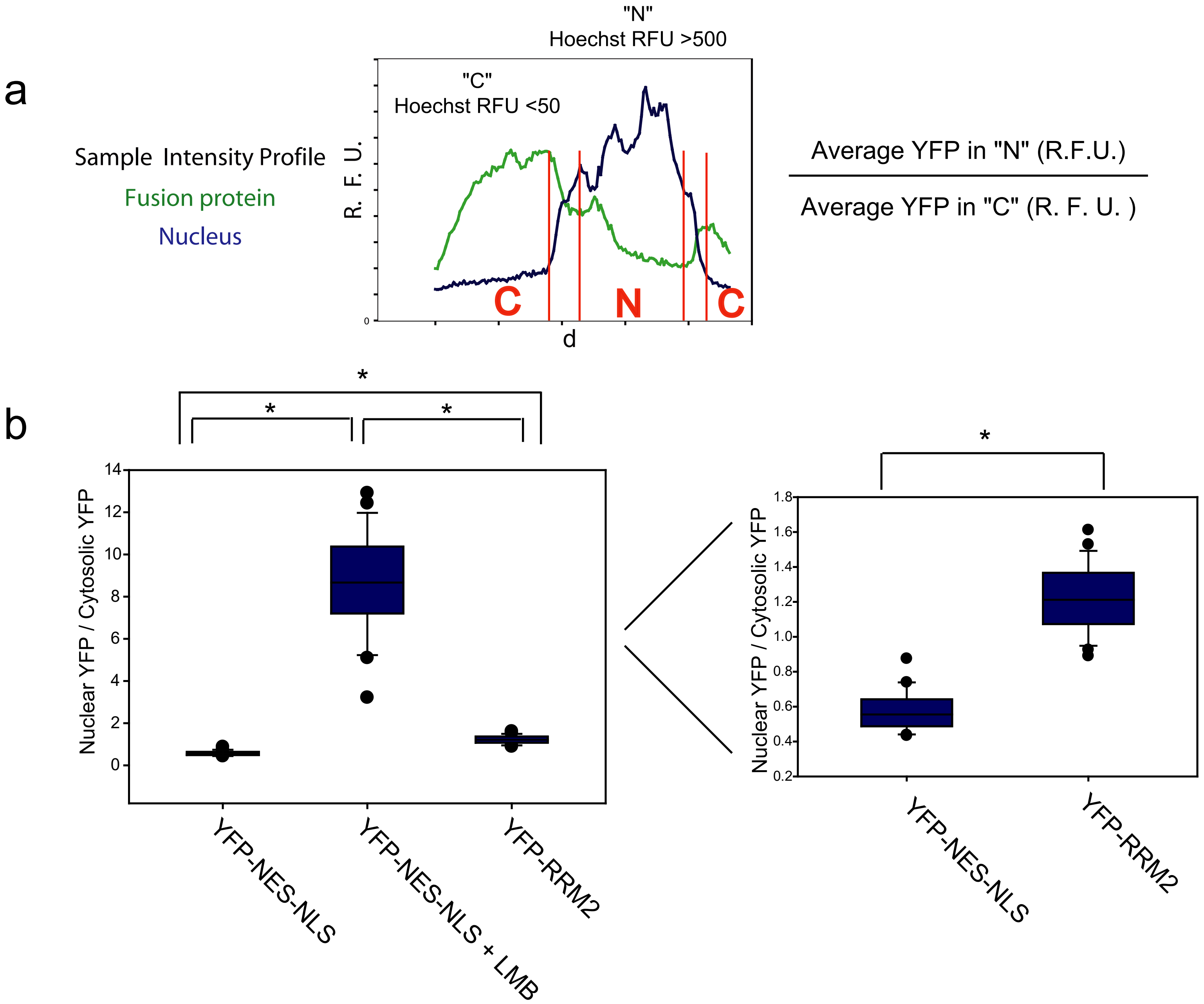

Supplementary Figure S1. Nuclear exclusion, nuclear inclusion, and distributed phenotypes can be quantified

a. Calculation of the "Nuclear YFP/Cytosolic YFP" based on a sample intensity profile.
b. Box and whiskers plot comparison of "Nuclear YFP/Cytosolic YFP" in HeLa cells (from Figure 2) transfected with the indicated YFP fusion protein. n=20 cells from two independent experiments.* represents p<0.001 in Mann-Whitney Rank Sum Test. Right: selected values plotted on smaller scale for direct comparison.

Supplementary Figure S2

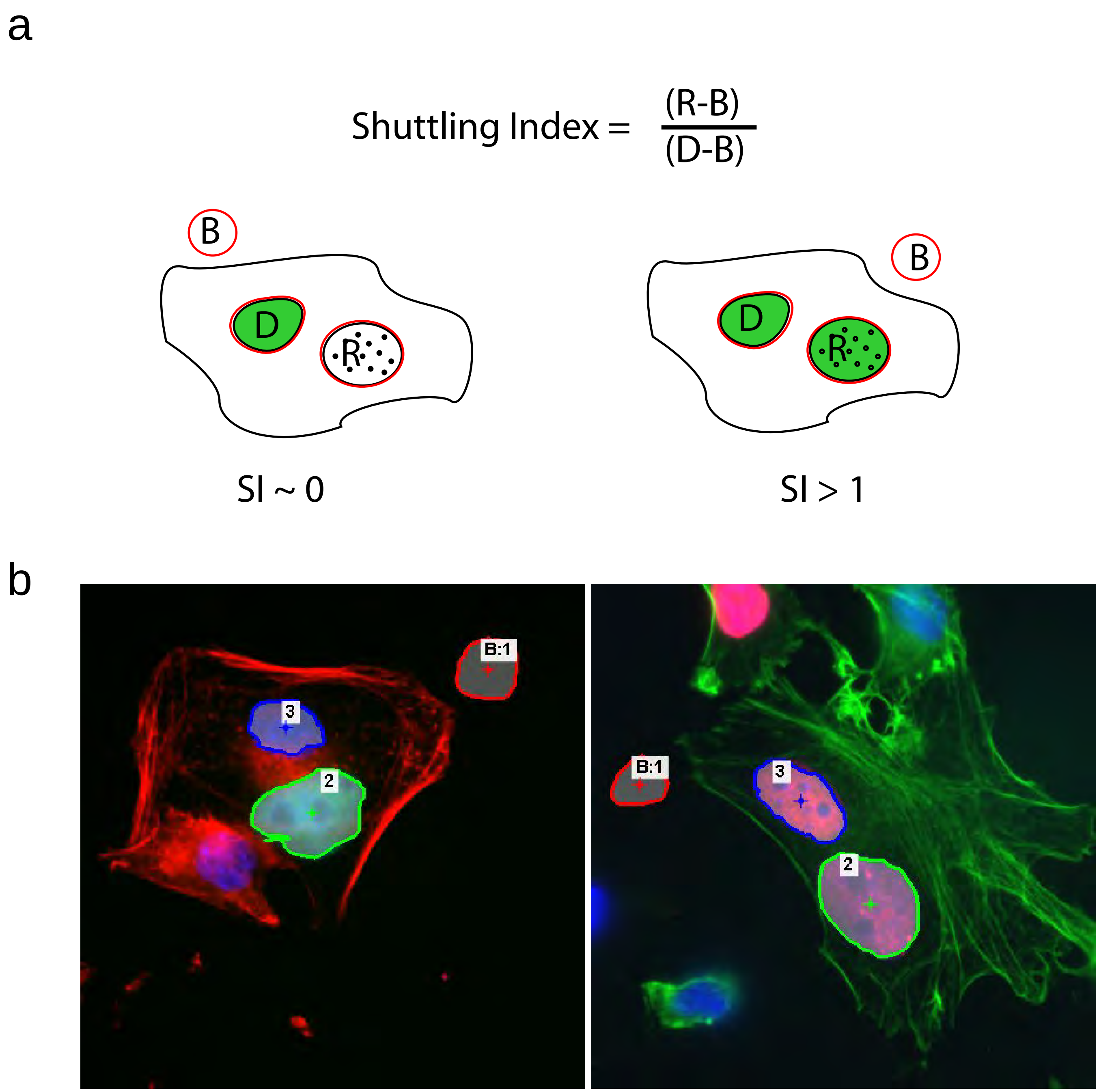

Supplementary Figure S2. Quantitation of Heterokaryon shuttling assays

a. Schematic demonstrating quantitation of heterokaryon shuttling assays.

b. Sample images from quantitation of heterokaryon shuttling assays. "B:1" : background fluorescence, "2" : donor nucleus (HeLa), "3": recipient nucleus (3T3). Shown in merged view: Left: Hoescht stain: blue, Actin skeleton: red, YFP-hnRNPC: green. Right: Hoescht stain: blue, Actin skeleton: green, Flag TDP-43: red.

Supplementary Figure S3

a

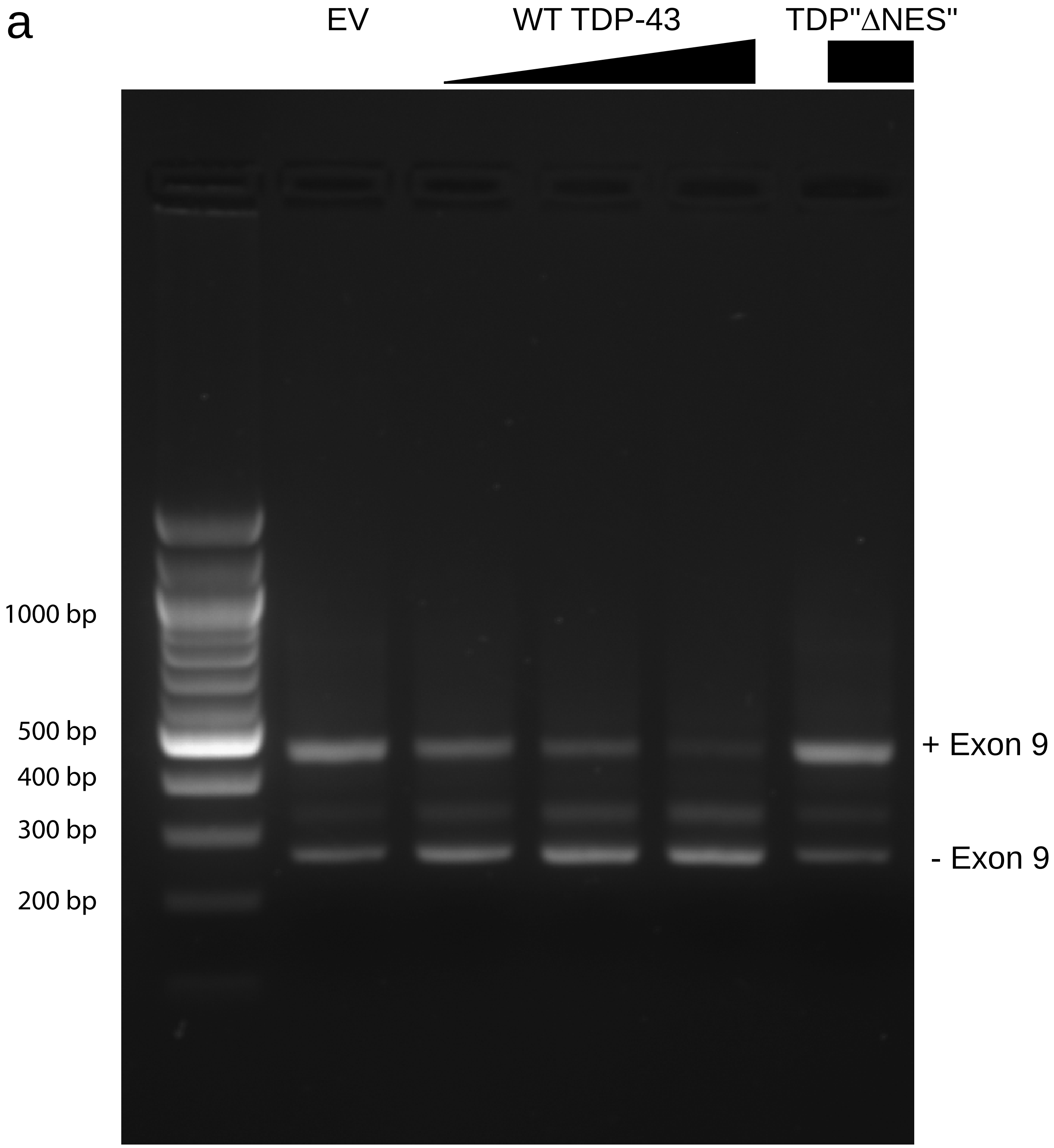

b

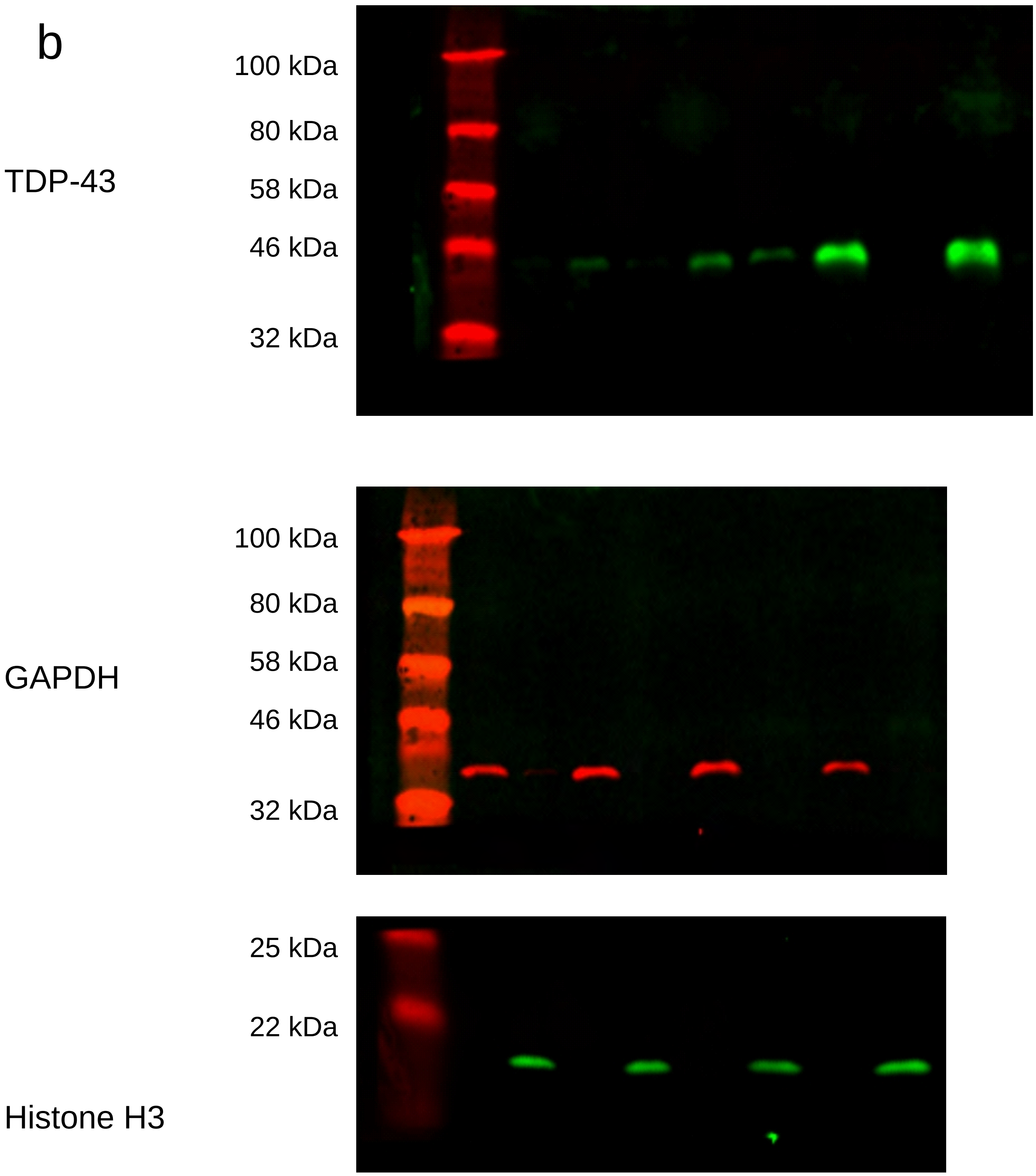

TDP-43
100 kDa
80 kDa
58 kDa
46 kDa
32 kDa
GAPDH
100 kDa
80 kDa
58 kDa
46 kDa
32 kDa
Histone H3
25 kDa
22 kDa

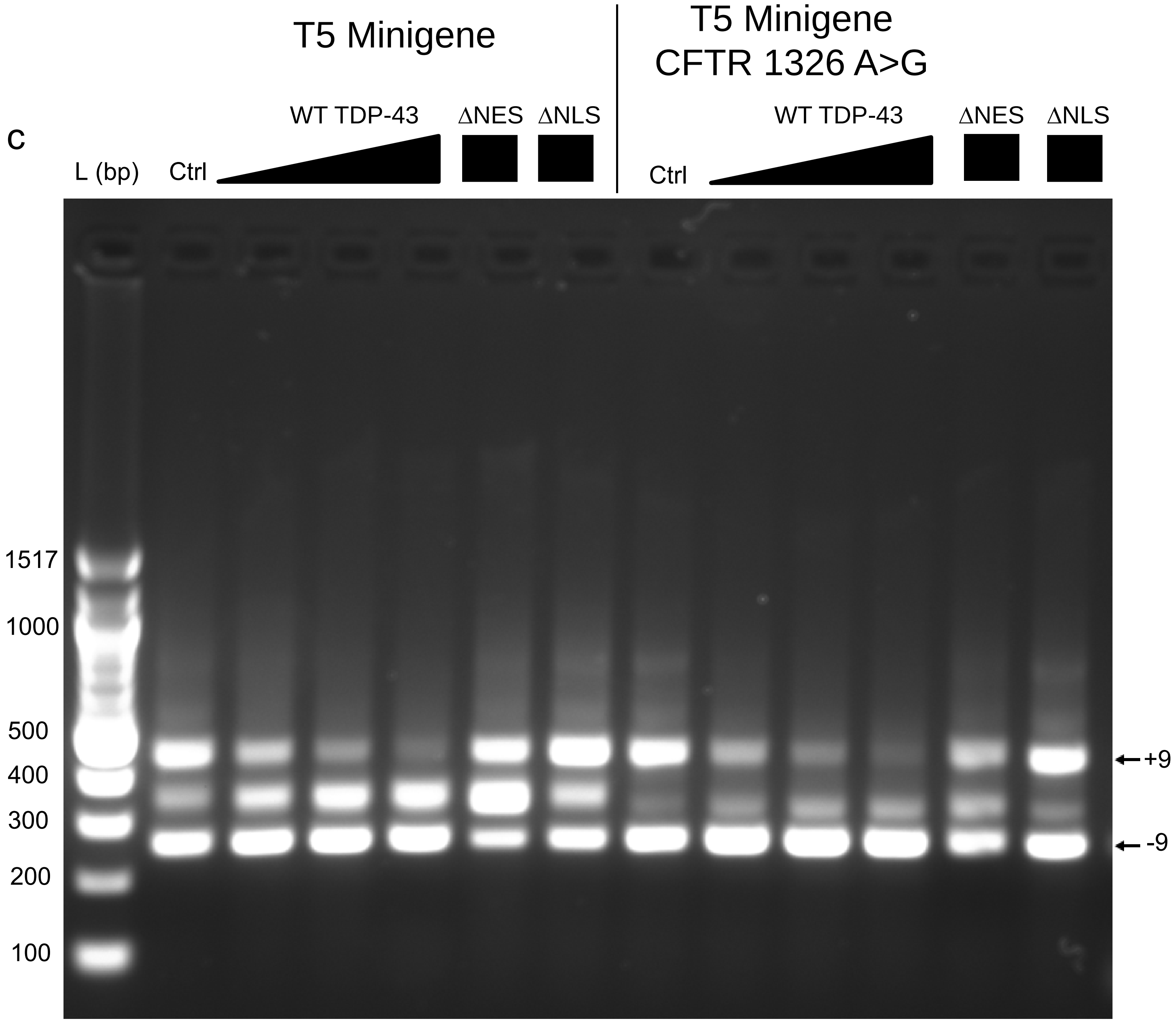

Supplementary Figure S3. CFTR T5 minigene with 1326A>G exhibits TDP-43 dependent CFTR exon 9 skipping.

- **a.** Uncropped gel from Figure 4a
- **b.** Uncropped images from Figure 4b. Western blot membrane was cut, and the top was probed for TDP-43 and GAPDH using different secondary antibodies. The bottom was probed for Histone H3.
- **c.** CFTR exon 9 splicing assay: HeLa cells were cotransfected with TDP-43 and CFTR T5 minigene (original or 1326 A>G). The "Ctrl" lane was cotransfected with empty vector cDNA and the CFTR T5 minigene. Cells in the three WT TDP-43 conditions were transfected with: 0.05 ug WT TDP-43 cDNA , 0.15 ug WT TDP-43 cDNA, and 0.5 ug WT TDP-43 cDNA. Cells in the mutant TDP-43 conditions were transfected with 0.5 ug TDP-43 cDNA. RNA was extracted and reverse-transcribed; cDNA was PCR amplified and run on an agarose gel. On the far left is the DNA ladder; bp stands for base pairs.

Supplementary Figure S4

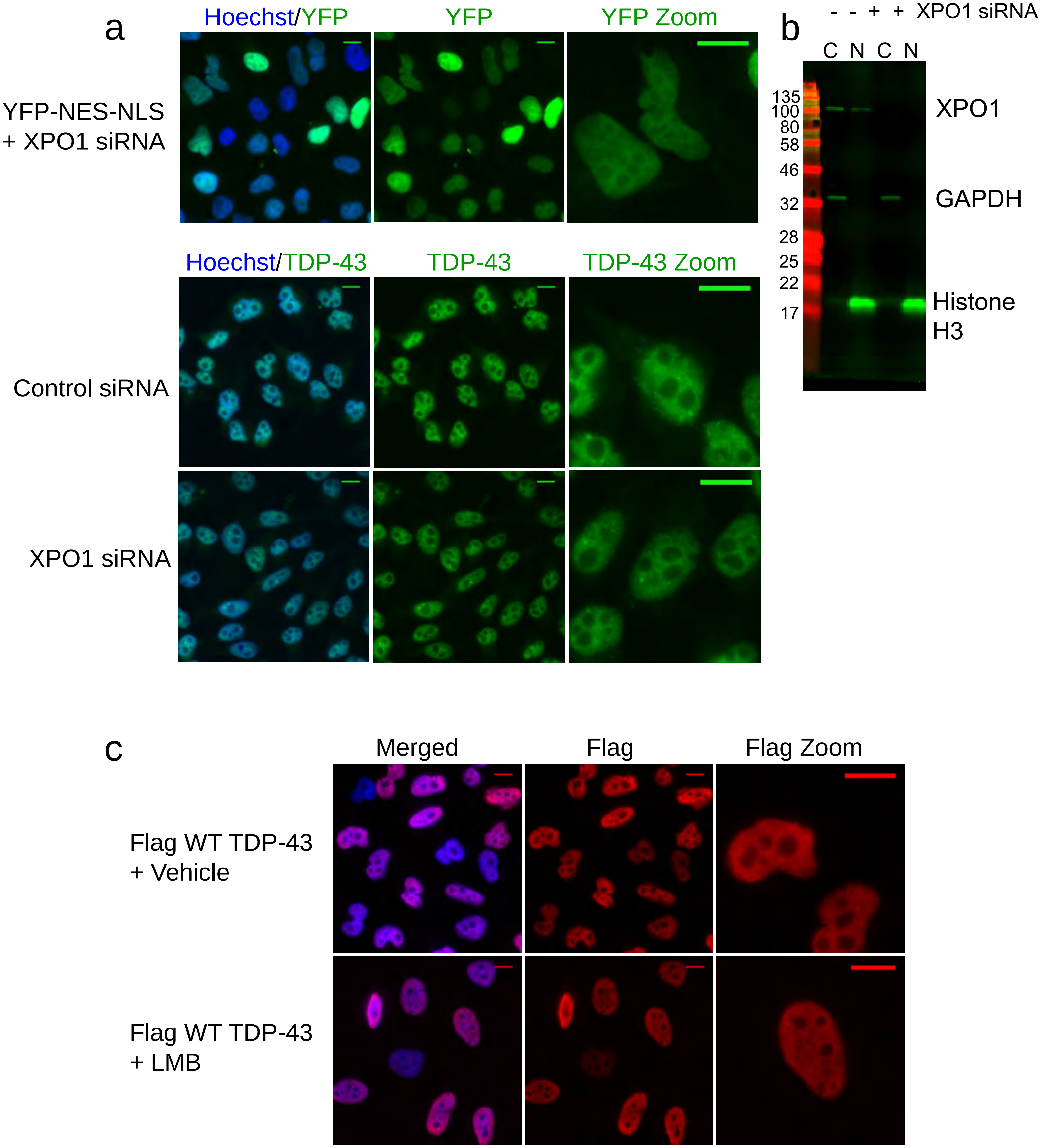

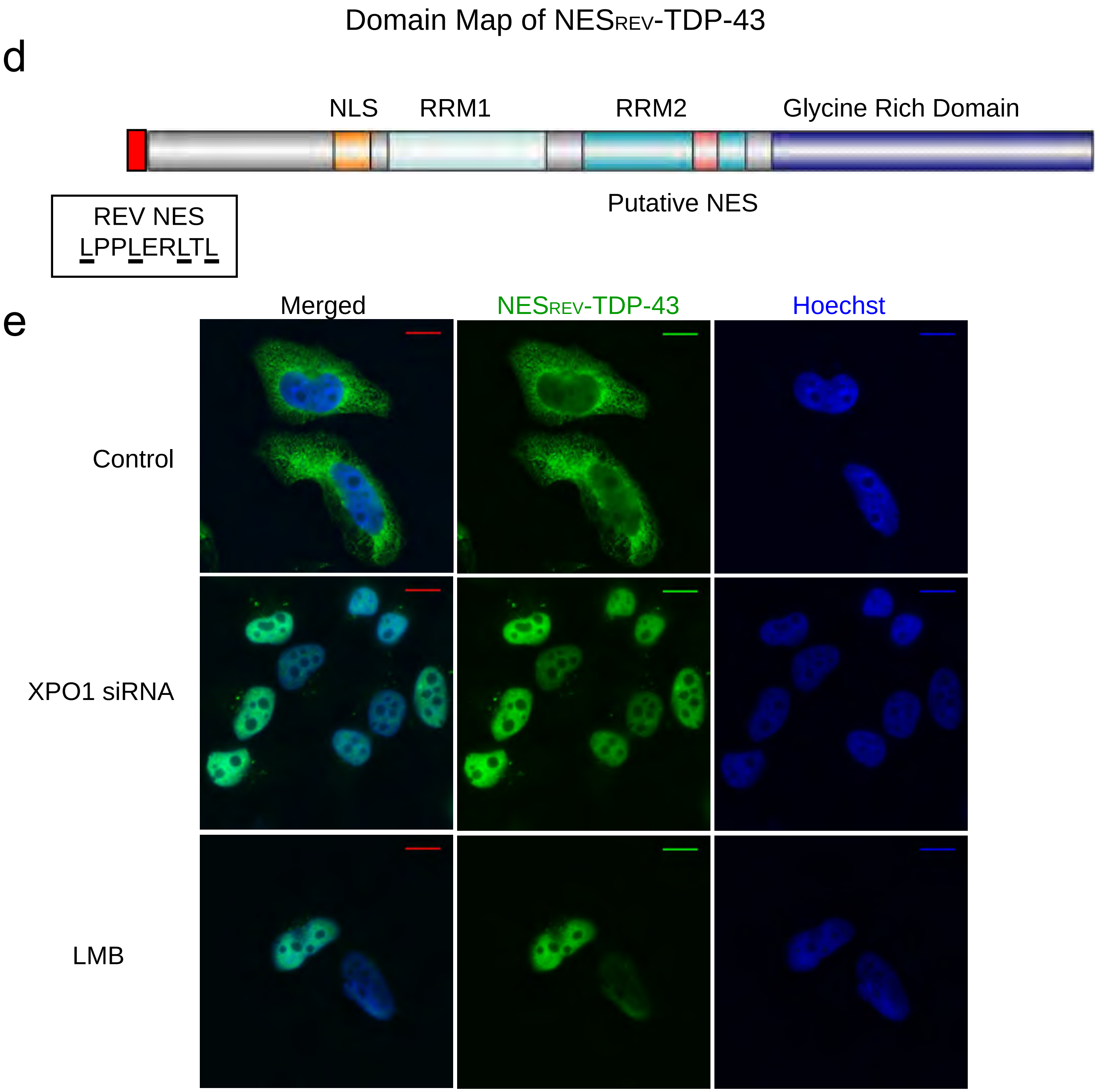

Supplementary Figure S4. WT TDP-43 is predominantly nuclear and does not contain a nuclear retention signal

a. Top: direct fluorescence of HeLa cells treated with XPO1 siRNA, expressing reporter YFP-NES$_{PKI}$-NLS$_{SV40}$. Bottom: Immunofluorescence of endogenous TDP-43 in HeLa cells treated with XPO1 siRNA.

b. Western blotting of Cytosolic "C" or Nuclear "N" lysates from HeLa cells treated with either control or XPO1 siRNA. XPO1, cytosolic control GAPDH and nuclear control Histone H3 shown. Ladder on left, molecular weight in kDa.

c. Immunofluorescence of HeLa cells expressing Flag WT TDP-43, in cells treated either by vehicle or Leptomycin B (LMB), 10 nM for 12 hours.

d. Domain map of fusion protein NES$_{REV}$-TDP-43. Peptide sequence of REV NES is detailed. Domain map formatted using IBS Cuckoo.[43]

e. Immunofluorescence of HeLa cells expressing fusion protein NES$_{REV}$-TDP-43, detected by TDP-43 antibody. Indicated cells were treated with XPO1 siRNA or Leptomycin B (LMB), 10 nM for 12 hours.

For a), c), and e): Nuclei are stained with Hoechst. Images are repesentative of 3 independent experiments. Scale bar- 10 um

Supplementary Figure S5

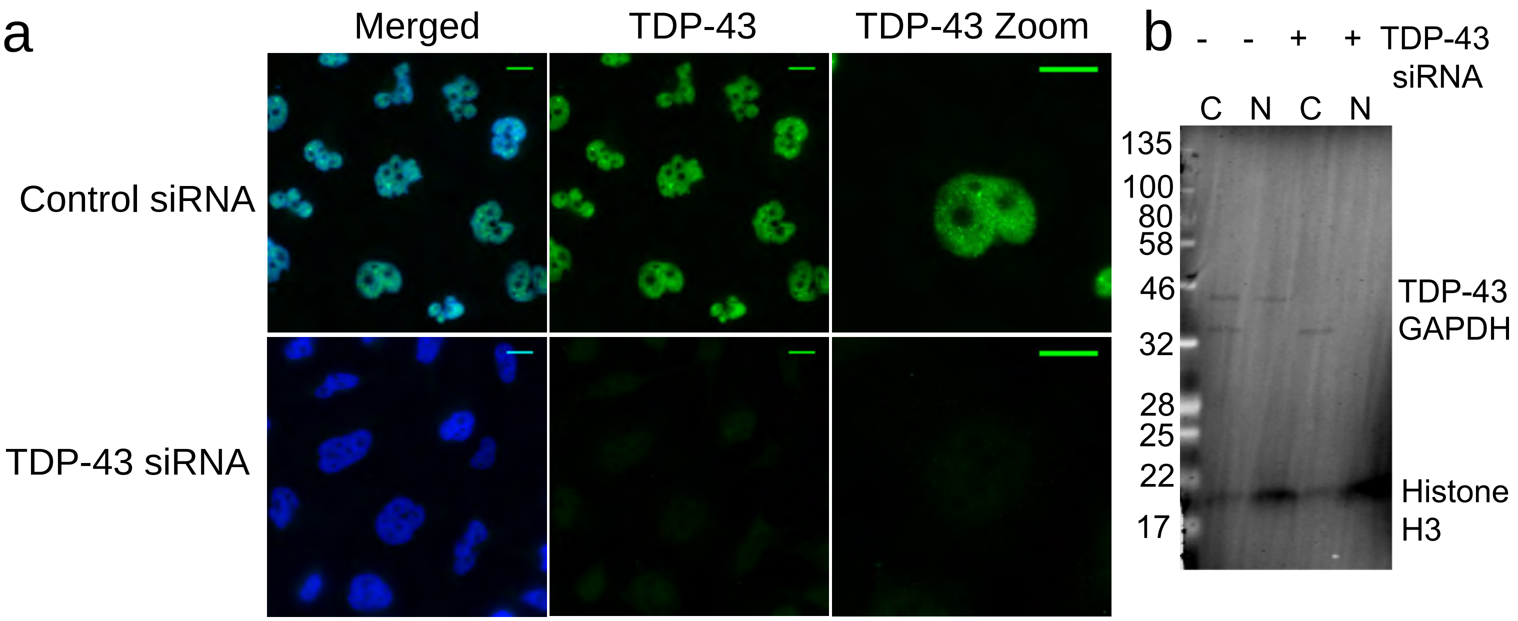

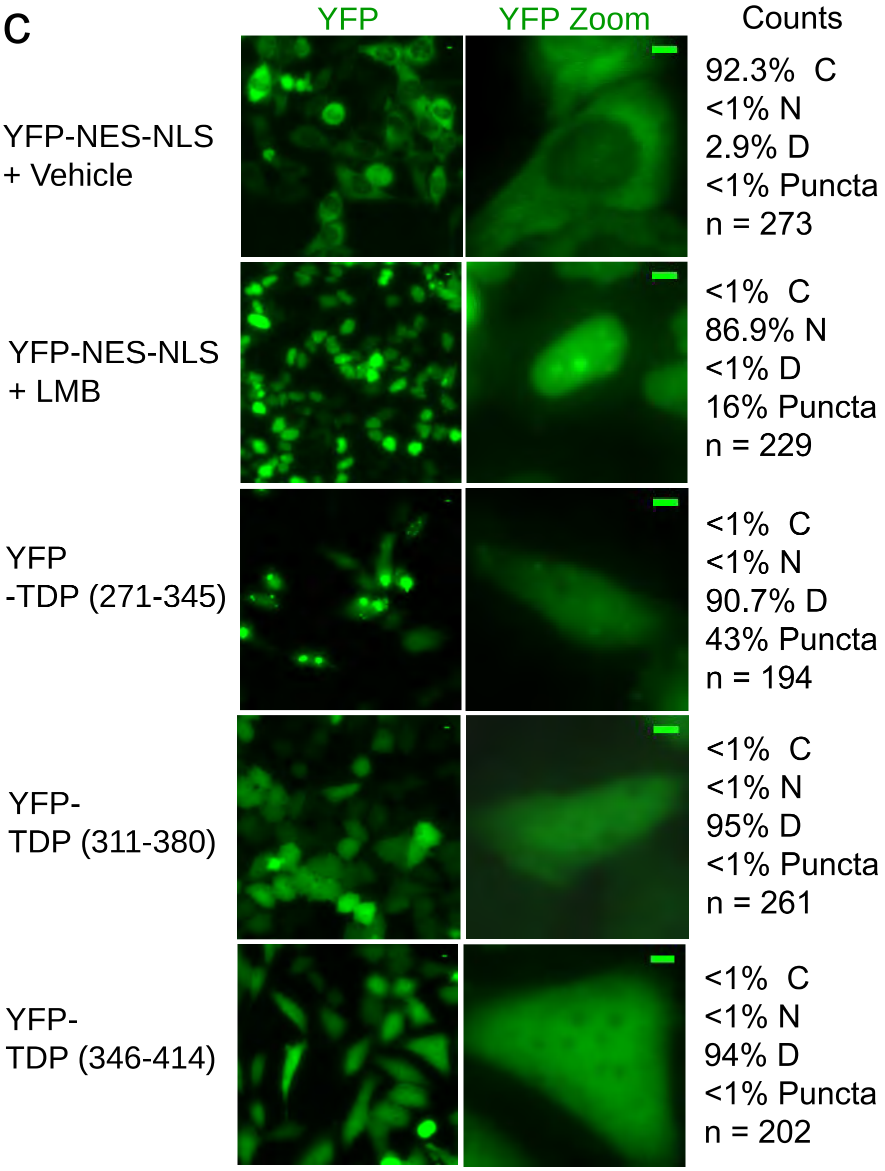

Supplementary Figure S5. Endogenous TDP-43 is efficiently depleted by TDP-43 siRNA, and C-terminus of TDP-43 does not contain a redundant NLS.

a. Immunofluorescence of endogenous TDP-43 in HeLa cells treated with either control siRNA or TDP-43 siRNA. Scale bars 10 um. Images are representative of 3 independent experiments
b. Western blotting of cytosolic "C" and nuclear "N" lysates of HeLa cells treated with control or TDP-43 siRNA. TDP-43, cytosolic control GAPDH, and nuclear control Histone H3 shown. Molecular ladder in kDa on left.
c. Direct fluorescence of HeLa cells expressing indicated fusion proteins. Indicated cells were treated with Leptomycin B (LMB), 10 nM for 12 hours. Scale bars 10 um. Images are representative of 3 independent experiments. Also shown: Percentages for 3 independent experiments: "N" nuclear YFP, "C" cytosolic YFP, "D" distributed YFP, "Puncta" containing visible puncta. Cells that were mitotic or apoptotic were not categorized, but included in total cell count.

Supplementary Figure S6

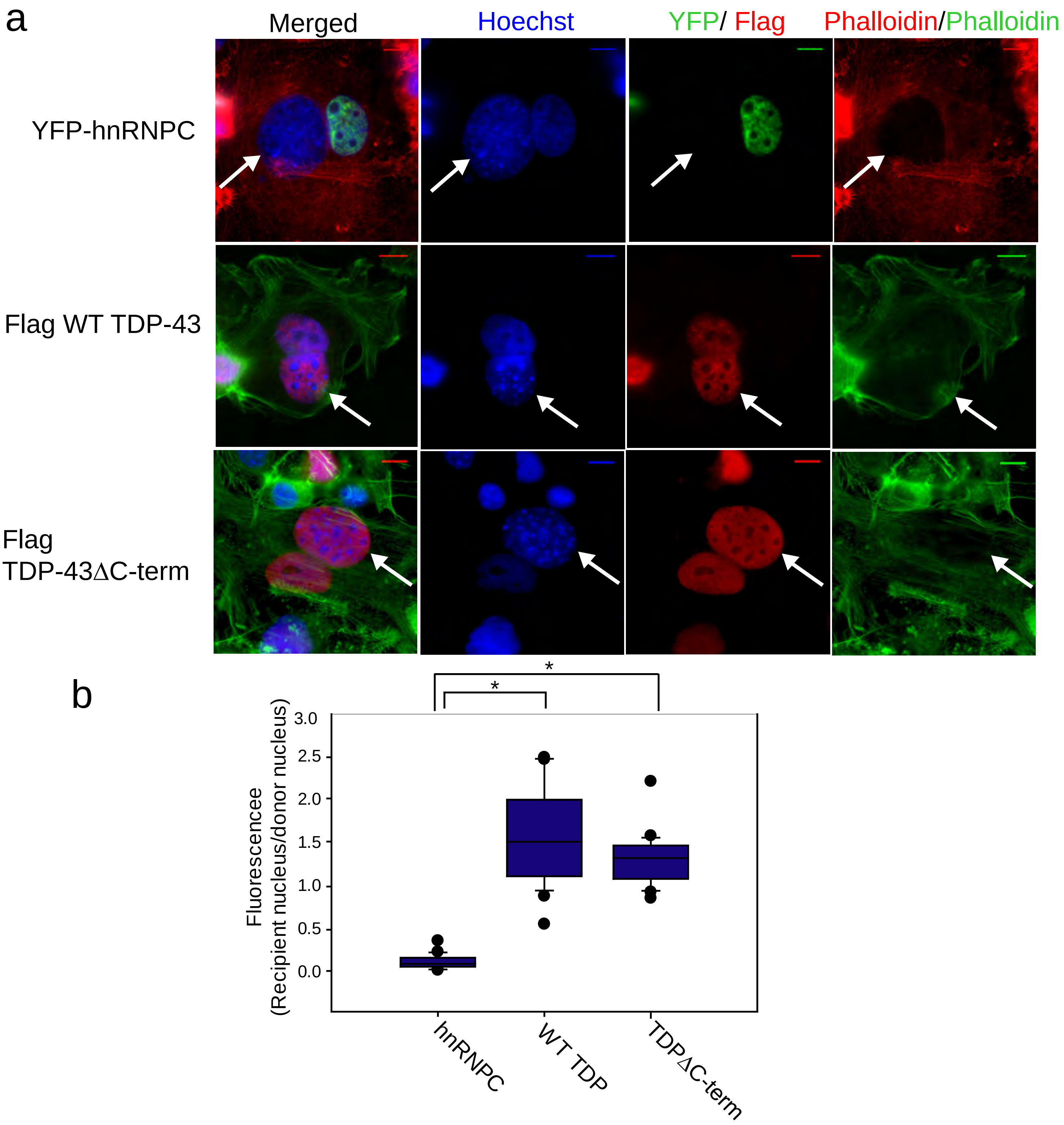

Supplementary Figure S6. C-terminus is not required for TDP-43 nuclear export

a. Example images from heterokaryon shuttling assay. YFP-hnRNPC was detected using direct fluorescence. Flag WT TDP-43 and Flag TDP-43 ΔC-terminus were detected using immunofluorescence with a Flag antibody. Nuclei were detected using Hoechst stain. Actin cytoskeleton was visualized using Phalloidin. Recipient nucleus indicated by arrow. Scale bars- 10um.

b. Quantitation of heterokaryon shuttling assay, as in Figure 3. Heterokaryons are pooled from 2 independent experiments. hnRNPC- 15 heterokaryons counted. Flag WT TDP-43- 22 heterokaryons counted. Flag TDP-43ΔC terminus- 18 heterokaryons counted. * indicates significant difference between groups, $p < 0.001$ using Mann-Whitney Rank Sum Test.
